# TRPA1 is essential for the vascular response to environmental cold exposure

**DOI:** 10.1038/ncomms6732

**Published:** 2014-12-11

**Authors:** Aisah A. Aubdool, Rabea Graepel, Xenia Kodji, Khadija M. Alawi, Jennifer V. Bodkin, Salil Srivastava, Clive Gentry, Richard Heads, Andrew D. Grant, Elizabeth S. Fernandes, Stuart Bevan, Susan D. Brain

**Affiliations:** 1 BHF Cardiovascular Centre of Excellence and Centre of Integrative Biomedicine, Cardiovascular Division, King’s College London, London SE1 9NH, UK; 2 Wolfson Centre for Age Related Diseases, King’s College London, London SE1 1UL, UK

## Abstract

The cold-induced vascular response, consisting of vasoconstriction followed by vasodilatation, is critical for protecting the cutaneous tissues against cold injury. Whilst this physiological reflex response is historic knowledge, the mechanisms involved are unclear. Here by using a murine model of local environmental cold exposure, we show that TRPA1 acts as a primary vascular cold sensor, as determined through TRPA1 pharmacological antagonism or gene deletion. The initial cold-induced vasoconstriction is mediated via TRPA1-dependent superoxide production that stimulates α_2C_-adrenoceptors and Rho-kinase-mediated MLC phosphorylation, downstream of TRPA1 activation. The subsequent restorative blood flow component is also dependent on TRPA1 activation being mediated by sensory nerve-derived dilator neuropeptides CGRP and substance P, and also nNOS-derived NO. The results allow a new understanding of the importance of TRPA1 in cold exposure and provide impetus for further research into developing therapeutic agents aimed at the local protection of the skin in disease and adverse climates.

## Abstract (web summary)

Blood flow in the skin of mammals changes in response to cold, but the mechanisms driving this response are unclear. Aubdool *et al*. show that the non-selective cation channel, TRPA1, is a vascular cold sensor and required for the vascular protective response to local cold exposure.

## Graphical abstract

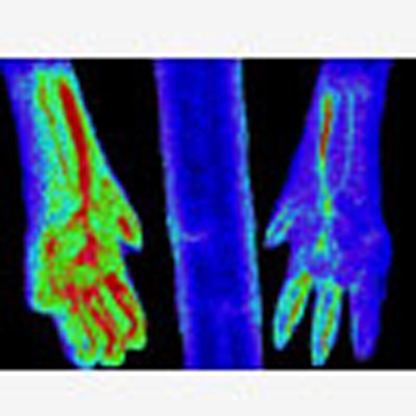

Mechanisms involved in the vascular response to cold have been under study for decades^1^. Local cold exposure in mammals leads to an initial, rapid-onset vasoconstriction that protects against heat loss and this is followed by recovery, involving vasodilation, which is essential to protect the area against local cold-induced injuries, such as chilblains and susceptibility to frostbite^1^,^2^,^3^. Mammals respond to cool temperatures with vasodilatation, which is associated with rewarming and a healthy peripheral vasculature^3^. A loss of cold-induced reflex recovery, associated with vasodilatation is a marker of peripheral vascular disease or injury, leading to painful conditions such as Raynaud’s disease^4^. Despite heavy debate, the mechanisms behind the mammalian cold-induced reflex remain unclear and the cutaneous thermosensitive components are unknown. Studies have focused on sympathetic constrictor mechanisms as a primary driver, with some evidence of sensory nerve involvement^5^.

We hypothesized that the cold (<17 °C) sensitive and non-selective cation channel, transient receptor potential ankyrin-1 (TRPA1) channel^6^, may play a pivotal physiological role in cold-induced vascular responses. The role of TRPA1 as a thermosensor in vascular responses is unexplored, although it has been shown to act as a cold sensor in Chinese Hamster Ovary cells in Ca^2+^ imaging studies^6^ and be involved in mediating cold-induced hyperalgesia in pathological states^7^,^8^,^9^,^10^. TRPA1 activation by a range of exogenous and endogenous mediators can occur by covalent activation of the cysteine residues localized to the amino terminus^11^. There is little information on the endogenous role of TRPA1 in cardiovascular regulation at present. Previous studies have shown that TRPA1 agonists, either the exogenous vegetable-derived agonist mustard-oil or the endogenous agonist 4-oxononenal (4-ONE), mediates cutaneous vasodilatation via the activation of sensory nerves, but the physiological relevance of this is unknown^12^,^13^,^14^. However, TRPA1-mediated constrictor responses have not been observed. TRPA1 agonists mediate dilation of peripheral resistance arteries *in vitro*, especially in vessels, such as the cerebral vasculature^15^,^16^.

The goal of this study is to determine whether TRPA1 plays a role as a vascular sensor of noxious cold, either initiating responses or acting downstream of other cold-sensing proteins. The experiments were designed using a novel murine *in vivo* model of local acute environmental cold exposure in skin. To achieve this, cutaneous blood flow was measured with a full-field laser perfusion imager (FLPI) *in vivo* in genetically modified mice and pharmacologically designed experiments. *Ex vivo* molecular and biochemical techniques were used to delineate the role of TRPA1.

## Results

### Local cold-induced vascular response is dependent on TRPA1

**Figure 1:**
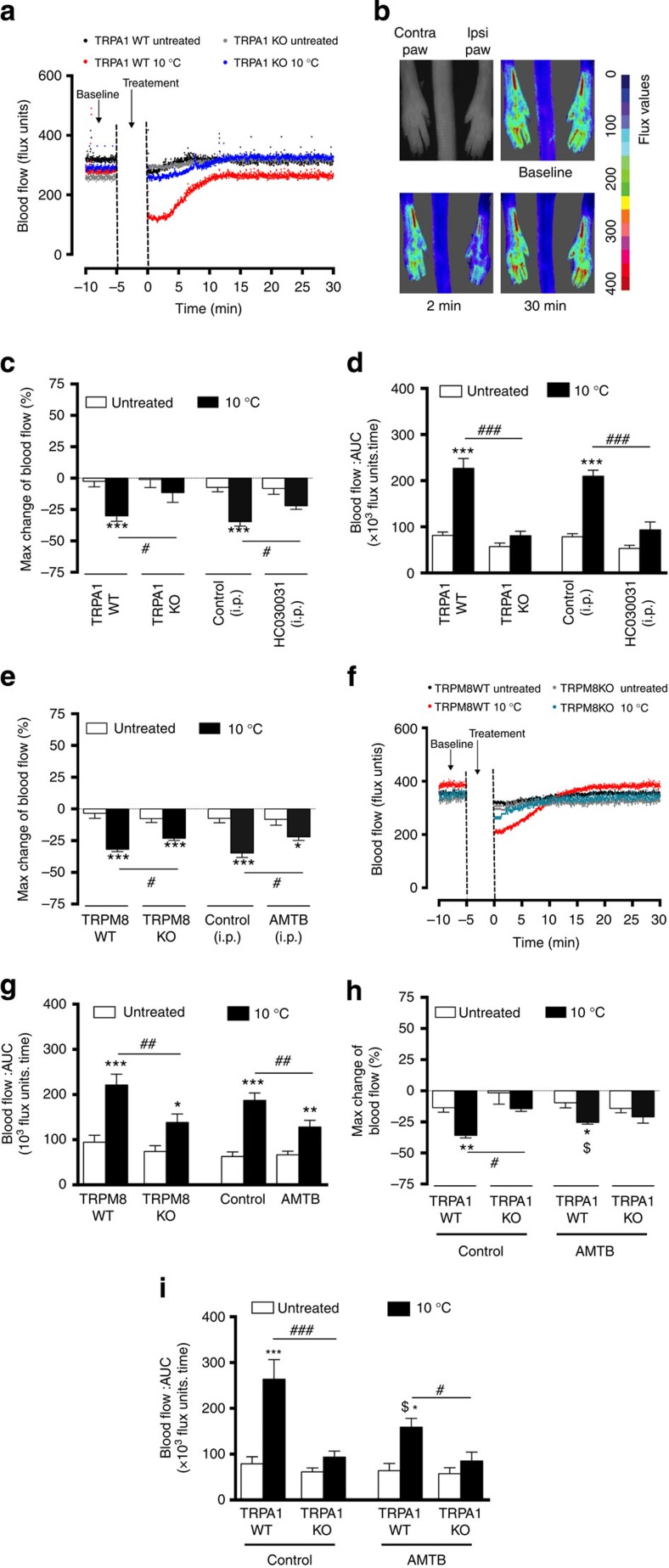
Cold-induced vascular response is dependent on TRPA1. Blood flow was measured using FLPI in anaesthetized mice following immersion of the ipsilateral hindpaw in cold (10 °C) water and contralateral paw remained untreated. (**a**) Representative blood flow trace of a cold-induced response in WT and TRPA1 KO mice. (**b**) Representative FLPI pictures alongside grey/black ‘photo’ showing blood flow at baseline, 2 and 30 min in cold-treated hindpaw. (**c**) % Change in hindpaw blood flow from baseline to 0–2 min following cold treatment (maximum vasoconstriction) and (**d**) Restoration of cutaneous blood flow, as assessed by AUC for 30 min following cold treatment in WT (*n*=12) and TRPA1 KO (*n*=9) or WT mice pretreated with TRPA1 antagonist HC030031 (100 mg kg^−1^, i.p., 30 min, *n*=10) or control (10% DMSO in saline, i.p., 30 min, *n*=8) before cold treatment. (**e**) % Maximum change in hindpaw blood flow from baseline to 0–2 min following cold treatment (maximum vasoconstriction) in WT (*n*=10) and TRPM8 KO (*n*=14) mice or WT mice pretreated with TRPM8 antagonist AMTB (10 mg kg^−1^, i.p., 30 min, *n*=8) or control (10% DMSO in saline, i.p., 30 min, *n*=10) before cold exposure. (**f**) Representative trace of a cold-induced response in WT and TRPM8 KO mice. (**g**) Restoration of cutaneous blood flow, assessed by AUC for 30 min following cold treatment in WT (*n*=10) and TRPM8 KO (*n*=14) mice or WT mice pretreated with TRPM8 antagonist AMTB (10 mg kg^−1^, i.p., 30 min, *n*=8) or control (10% DMSO in saline, i.p., 30 min, *n*=10) before cold treatment. (**h**) % Change in hindpaw blood flow from baseline to 0–2 min following cold treatment (maximum vasoconstriction) and (**i**) Restoration of cutaneous blood flow, assessed by AUC for 30 min following cold treatment in TRPA1 WT (*n*=5) and KO (*n*=5) pretreated with control (10% DMSO in saline, i.p., 30 min) or TRPA1 WT (*n*=4) and KO (*n*=4) pretreated with AMTB (10 mg kg^−1^, i.p., 30 min). All error bars indicate s.e.m. *P<0.05, **P<0.01, ***P<0.001 versus respective untreated, ^#^*P*<0.05, ^##^*P*<0.01, ^###^*P*<0.001 versus cold-treated, $*P*<0.05 versus cold-treated TRPA1 WT (analysis of variance, Bonferroni *post hoc* test).

The cold model was developed and characterized in male anaesthetized wild-type (WT) mice (8–12 weeks). Following baseline blood flow measurements, the ipsilateral hindpaw was immersed in cold water (10 °C for 5 min), whilst the contralateral paw remained untreated at room temperature. Exposure to temperatures from 4 to 23 °C (Supplementary Table 1) revealed that the vasoconstriction response to 10 °C exhibited substantial TRPA1 dependency. Blood flow was then assessed immediately following cooling, for 30 min using FLPI, to allow dynamic measurement, at a time period chosen to ensure the response to cold exposure was complete (Fig. 1a). The maximum vasoconstriction was observed at 0 to 2 min following local cooling and determined as the % maximum decrease in blood flow from the precooling baseline (Fig. 1a–c and Supplementary Fig. 1). This response was substantially less in TRPA1 knockout (KO) mice and in WT mice pretreated with the TRPA1 antagonist HC030031 (ref. 17) (Fig. 1c). It was not technically feasible to measure blood flow with the FLPI during cold (10 °C) water immersion. However, an increased clearance, indicative of active constriction, was measured by ^99m^Technetium clearance during cooling and this response was not observed in the presence of the TRPA1 antagonist (Supplementary Fig. 2). WT and TRPA1 KO mice have similar cardiovascular parameters at baseline (Supplementary Fig. 3) and there was no significant change in vascular responses to immersion in 26 °C water (Supplementary Table 1). Thus, TRPA1 mediates the initial vasoconstrictor response of the local cold-induced vascular response. The restorative response involves vascular relaxation, which follows the constrictor phase and is measured as area under the curve (AUC) (Fig. 1a,d and Supplementary Fig. 1). This response involves blood flow recovery to baseline levels, referred to as the ‘restorative phase’ of the cold-induced vascular response.

There is debate and conflicting evidence concerning the relative importance of TRPA1 as a primary cold sensor in cells and *in vivo* models. TRPM8 (sensitive at temperatures <25 °C (ref. 17)) rather than TRPA1 is considered to have a major role in the response to deep body cooling^18^ and in cold-induced pain behaviours^19^. Here the cold-induced vasoconstriction was partially but significantly reduced in TRPM8 KO mice or in WT mice pretreated with the TRPM8 antagonist AMTB compared with control groups (Fig. 1e,f). AMTB pretreatment did not further modify the cold-induced vascular response in TRPA1 KO mice (Fig. 1h) and hence these results indicate that TRPM8 is involved, but played a secondary role to TRPA1 in the cold-induced vascular response in this study. Whilst the TRPA1 antagonist HC030031 abolished vasodilation induced by the exogenous TRPA1 agonist cinnamaldehyde in the mouse ear, AMTB had no effect (Supplementary Fig. 4A–C), suggesting that the pharmacological blockade of TRPM8 does not influence the vascular effects of TRPA1 to other agonists. The results for the restorative increased blood flow response following local cold exposure (Fig. 1f,g,i) show a return to baseline levels in each case, the magnitude of which correlates with that of the vasoconstrictor response. TRPA1 is co-expressed in 60–75% of TRPV1-expressing sensory C-fibre nerves^6^,^20^. However, neither pretreatment with the TRPV1 antagonists SB366791, AMG9810 nor gene deletion of TRPV1 altered cold-induced vascular responses (Supplementary Table 2). Mice with TRPV4 gene deletion (a predicted warm temperature sensor of 25–34 °C) (refs 21, 22) were similarly not affected (Supplementary Table 2).

### α_2C_-adrenoceptor and superoxide in cold treated hindpaws

To investigate whether TRPA1 could stimulate the initial vasoconstrictor phase via noradrenaline release from sympathetic nerves, noradrenaline concentration was measured in cold-treated and untreated-hindpaw tissue samples collected at the peak of the cold-induced vasoconstriction. Tissue noradrenaline concentration was significantly decreased in the WT cold-treated hindpaw, as assessed by enzyme linked immuno sorbent assay (ELISA). This is possibly as a consequence of the intense vasoconstriction and short half-life of noradrenaline. The noradrenaline concentrations were unchanged in the cold-treated hindpaw of TRPA1 KO (Supplementary Fig. 5).

**Figure 2:**
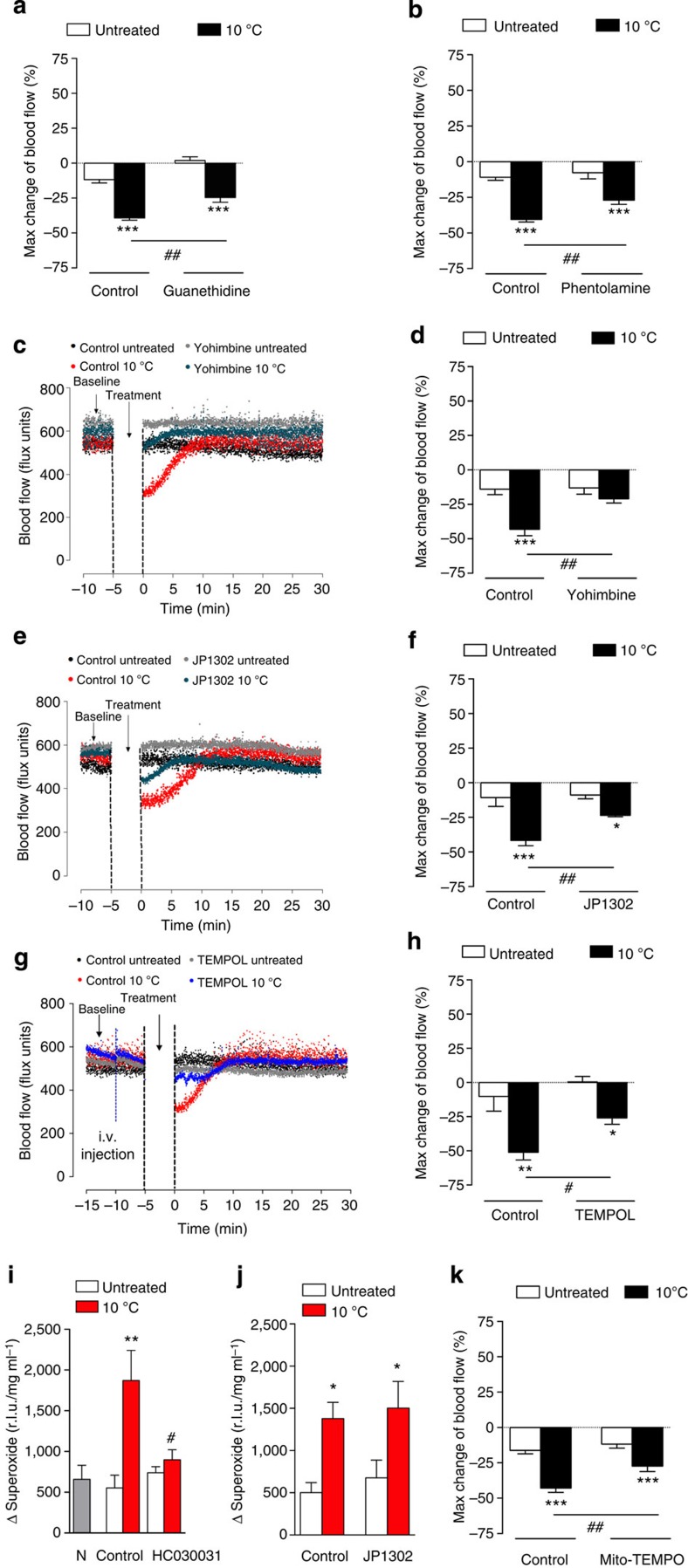
Sympathetic nerves, α_2C_-adrenoceptors and superoxide in cold-induced vasoconstriction. Blood flow was measured using FLPI in anaesthetized mice following immersion of the ipsilateral hindpaw in cold (10 °C) water and the contralateral hindpaw remained untreated. (**a**) % Maximum change in hindpaw blood flow from baseline to 0–2 min following cold treatment (maximum vasoconstriction) in WT mice pretreated with guanethidine (30 mg kg^−1^, s.c., 4 days, *n*=7) or control (saline, s.c., 4 days, *n*=7) and (**b**) WT mice pretreated with the α-adrenoceptor antagonist phentolamine (10 mg kg^−1^, i.v., *n*=6) or control (saline, i.v., *n*=6). (**c**) Representative trace of a cold-induced response and (**d**) % Change in hindpaw blood flow from baseline to 0–2 min following cold treatment (maximum vasoconstriction) in WT mice treated with the α_2_-adrenoceptor antagonist yohimbine (10 mg kg^−1^, s.c., 30 min, *n*=5) or control (saline, s.c., 30 min, *n*=5). (**e**) Representative trace of a cold-induced response and (**f**) % Change in hindpaw blood flow from baseline to 0–2 min following cold treatment (maximum vasoconstriction) in WT mice treated with the α_2C_-adrenoceptor antagonist JP1302 (3 μg kg^−1^, s.c., 60 min, *n*=6) or control (saline, s.c., 60 min, *n*=6). (**g**) Representative blood flow trace of a cold-induced response and (**h**) % Change in blood flow from baseline to 0–2 min following cold treatment (maximum vasoconstriction) in WT mice treated with the SOD mimetic TEMPOL (30 mg kg^−1^, i.v., 5 min, *n*=4) or control (saline, i.v., 5 min, *n*=4). Superoxide levels were measured at 2 min (maximum vasoconstriction) following cold treatment in the hindpaw tissue samples by lucigenin chemiluminescence in (**i**) naïve, WT mice pretreated with TRPA1 antagonist HC030031 (100 mg kg^−1^, i.p., 30 min) or control (10% DMSO in saline, i.p., 30 min, *n*=4–5) and (**j**) WT mice pretreated with JP1302 (3 μg kg^−1^, s.c., 60 min) or control (saline, s.c., 30 min, *n*=6–8). (**k**) % Change in blood flow from baseline to 0–2 min following cold treatment (maximum vasoconstriction) in WT mice treated with the mitochondria-targeted superoxide scavenger mito-TEMPO (10 mg kg^−1^, i.p., 60 min, *n*=9) or control (saline, i.p., 60 min, *n*=8). All error bars indicate s.e.m. *P<0.05, **P<0.01,***P<0.001 versus respective untreated, ^#^*P*<0.05, ^##^*P*<0.01 versus cold-treated hindpaw (analysis of variance, Bonferroni *post hoc* test).

Furthermore, guanethidine-induced depletion of the peripheral sympathetic nervous system or the pretreatment with the α-adrenoceptor antagonist phentolamine caused a partial but significant effect on the cold-induced vasoconstriction (Fig. 2a,b). However, α_2C_-adrenoceptors have been shown to influence cold-induced vascular responses^23^,^24^, and pretreatment with the non-selective α_2_-adrenoceptor antagonist yohimbine or the selective α_2C_-adrenoceptor antagonist JP1302 also markedly attenuated the initial cold-induced vasoconstriction phase (Fig. 2c–f).

α_2C_-adrenoceptors are silent at 37 °C, but in response to cooling, their activity increases as a result of increased reactive oxygen species generation^25^,^26^. Pretreatment with the highly potent, cell-permeable superoxide dismutase (SOD) mimetic TEMPOL also significantly reduced local cold-induced vasoconstriction (Fig. 2g,h). To determine whether TRPA1 activation induced superoxide release, superoxide levels were measured using the Lucigenin luminescence assay in hindpaw skin tissues collected at the maximum vasoconstrictor phase. The results showed a significant increase in the total superoxide released in the cold-treated hindpaw compared with the untreated or naïve hindpaw at peak vasoconstriction following local cold treatment in WT mice, with no significant changes in WT mice pretreated with HC030031 (Fig. 2i) or TRPA1 KO mice (Supplementary Fig. 6). By comparison, JP1302 had no effect on superoxide generation (Fig. 2j). Thus, TRPA1 activation is linked to superoxide generation and in turn, α_2C_-adrenoceptor activation. The constrictor response to TRPA1 was significantly inhibited by mito-TEMPO implicating mitochondria as the possible source of superoxide generation (Fig. 2k).

**Figure 3:**
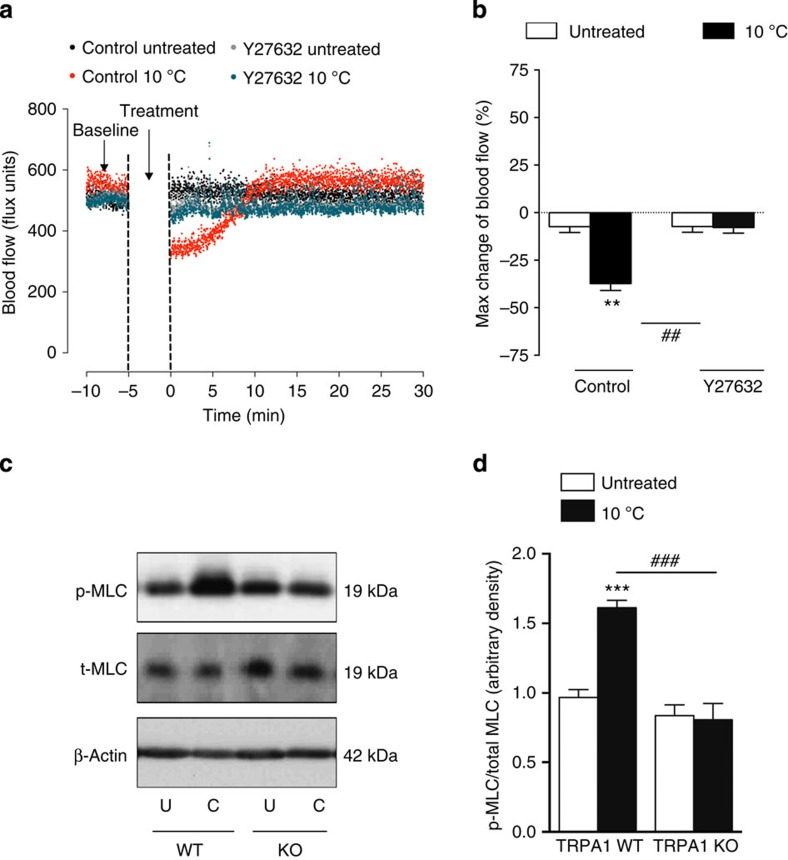
Rho-kinase-mediated MLC signalling in the cold-induced vasoconstriction. Blood flow was measured using FLPI in anaesthetized mice following immersion of the ipsilateral hindpaw in cold (10 °C) water and the contralateral hindpaw remained untreated. (**a**) Representative blood flow trace of a cold-induced vascular response in WT mice pretreated with the selective Rho-kinase associated protein kinase inhibitor Y27632 or control (saline). (**b**) % Maximum change in hindpaw blood flow from baseline to 0–2 min following local cold treatment (maximum vasoconstriction) in WT mice pretreated with Y27632 (5 mg kg^−1^, i.p., 30 min, *n*=6) and control (saline, i.p., 30 min, *n*=5). (**c**) Representative western blot of p-MLC at Ser^19^ of MLC and total MLC protein expression (uncropped blots are presented in Supplementary Figure 12). (**d**) Densitometric analysis of p-MLC and total MLC, normalized to β-actin (*n*=6). All error bars indicate s.e.m. **P<0.01, ***P<0.001 versus respective untreated, ^##^*P*<0.01, ^###^*P*<0.001 versus cold-treated hindpaw (analysis of variance, Bonferroni *post hoc* test).

The α_2C_-adrenoceptor mediated constriction is known to be highly dependent on Rho-associated kinase (Rho-kinase) activity^25^. Pretreatment with the potent, cell-permeable and selective Rho-kinase inhibitor Y27632 abolished the vasoconstrictor component of the local cold-induced vascular response in WT mice (Fig. 3a,b). The Rho-kinase-mediated myosin light chain (MLC) activation signalling pathway is involved in mediating the constriction of vascular smooth muscle cells^27^. Here there was a significant increase in the phosphorylation of MLC protein at Ser^19^ in hindpaw tissue samples at peak vasoconstriction following local cold treatment compared with untreated control tissues (Fig. 3c,d). This response was only observed in WT mice and not TRPA1 KO mice, suggesting an important role of MLC in regulating TRPA1-mediated responses induced by cold treatment in the maximum vasoconstriction phase.

### Restoration of blood flow post cold exposure requires TRPA1

The initial TRPA1-dependent cold-induced vasoconstriction directly precedes the recovery/restorative component (Fig. 1a,b), observed as a dilator response and analysed as AUC from the maximum of the vasoconstriction phase until the end of the measurement period (30 min; Fig. 1d). A highly significant restoration of blood flow was observed in control-treated groups, but not in the absence of the initial constriction response to cold (Fig. 1d). Therefore, neither WT mice treated with HC030031 (intraperitoneally, i.p. 30 min) before local cold treatment, nor TRPA1 KO mice (Fig. 1d) exhibited this restorative vascular relaxation. Indeed, as this restorative response was not observed in the absence of cold-induced vasoconstriction, it was impossible to assess from these experiments whether TRPA1 was involved in the recovery/dilator component.

**Figure 4:**
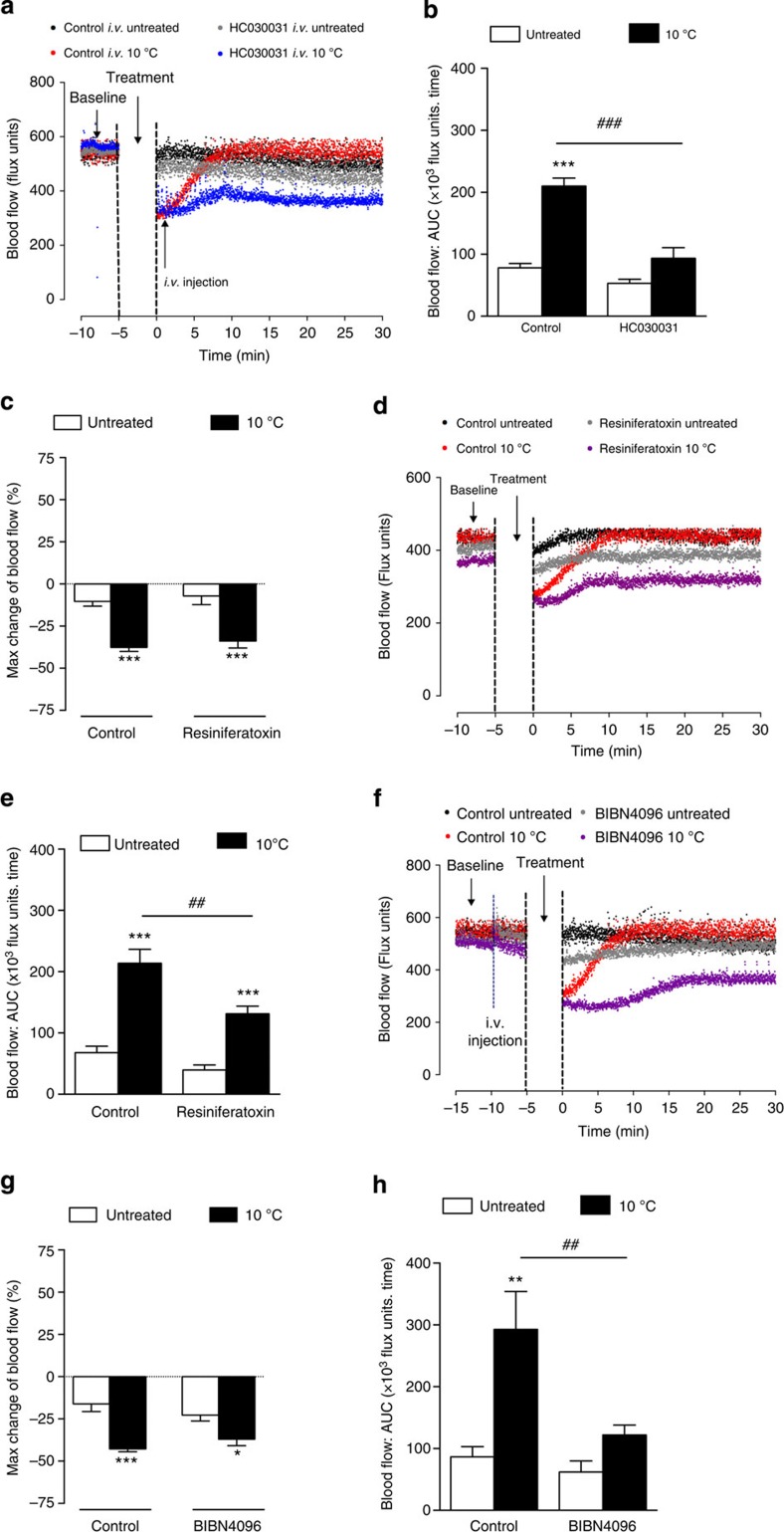
TRPA1 mediates the vasodilator component of cold-induced responses. Blood flow was measured using FLPI in anaesthetized mice following immersion of the ipsilateral hindpaw in cold (10 °C) water and the contralateral hindpaw remained untreated. (**a**) Representative blood flow trace of a cold-induced vascular response and (**b**) restoration of cutaneous blood flow, as assessed by AUC for 30 min following cold water immersion in WT mice pretreated with the TRPA1 antagonist HC030031 (6 mg kg^−1^, i.v., *n*=6) or control (8% DMSO in 2% Tween-80 in saline, *n*=6) at the maximum vasoconstriction phase following local cold water immersion. (**c**) % Maximum change in hindpaw blood flow from baseline to 0–2 min following local cold treatment (maximum vasoconstriction), (**d**) Representative blood flow trace of a cold-induced vascular response and (**e**) Restoration of cutaneous blood flow, as assessed by AUC for 30 min following cold water immersion in WT mice pretreated with the TRPV1 agonist resiniferatoxin (0.3 mg kg^−1^, s.c. daily., 4 days, *n*=8) or control (10% ethanol, 10% Tween-80 in saline, s.c. daily, 4 days, *n*=9). (**f**) Representative blood flow trace of a cold-induced vascular response, (**g**) % Maximum change in hindpaw blood flow from baseline to 0–2 min following local cold treatment (maximum vasoconstriction) and (**h**) Restoration of cutaneous blood flow, as assessed by AUC for 30 min following cold water immersion in WT mice pretreated with the CGRP receptor antagonist BIBN4096 (0.3 mg kg^−1^, i.v., 5 min, *n*=9) or control (saline, i.v., 5 min, *n*=10). All error bars indicate s.e.m. *P<0.05, **P<0.01, ***P<0.001 versus respective untreated, ^##^*P*<0.01, ^###^*P*<0.001 versus cold-treated hindpaw (analysis of variance, Bonferroni *post hoc* test).

Thus, we questioned whether TRPA1 activity was required to initiate the secondary restorative vasodilator phase by examining WT mice treated with the TRPA1 antagonist HC030031 (intravenously, i.v.) during the vasoconstriction phase, following local cold exposure. The results showed that HC030031, administered only once the vasoconstriction had occurred, prevented vasodilatation and as a consequence the return of blood flow to baseline. However, the vasoconstrictor response occurred normally in the respective control groups (Fig. 4a,b). Hence, TRPA1 is pivotal in initiating the vasodilator response, in addition to the vasoconstrictor response following cold exposure. In support of this, chronic treatment with the ultra-potent capsaicin analogue resiniferatoxin to remove the cutaneous sensory nerve component had no effect on the constrictor component (Fig. 4c,d), but significantly inhibited the dilator component, implicating the involvement of sensory neurogenic vasodilation (Fig. 4d,e and Supplementary Fig. 7). The role of TRPA1 as an activator of the sensory neuronal vasodilator axes was further implicated by the finding that pretreatment with the CGRP receptor antagonist BIBN4096 only attenuated the vasodilator component, whilst having no influence on the constrictor component (Fig. 4f,h).

### Neuropeptides and nitric oxide in the vasodilator component

**Figure 5:**
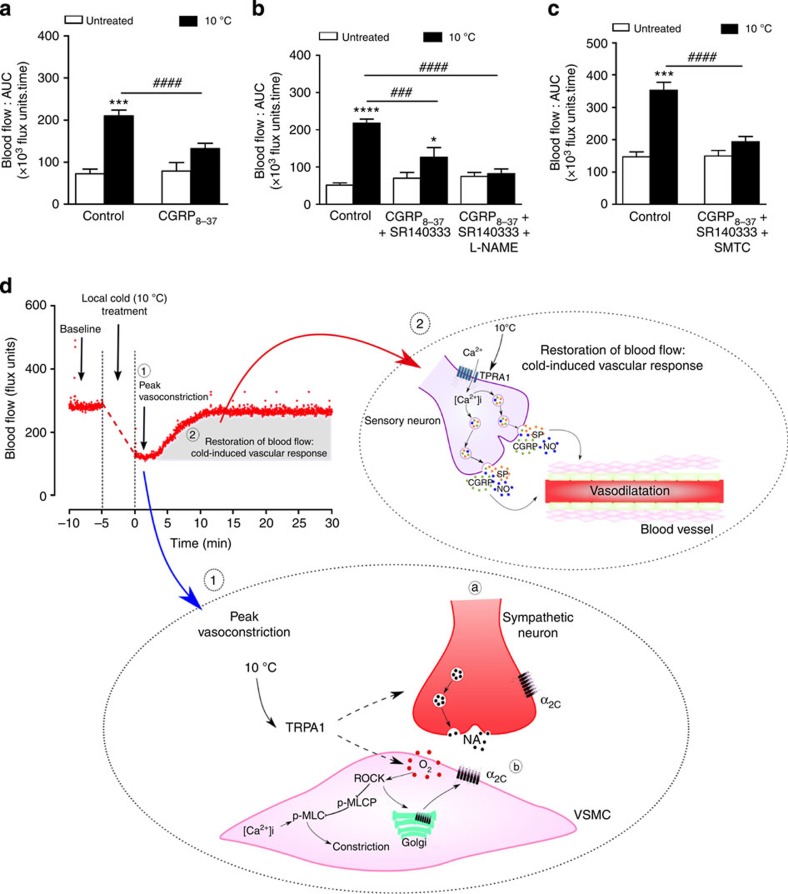
The cold-induced vascular response is dependent on neuropeptides. Blood flow was measured using FLPI in anaesthetized mice following immersion of the ipsilateral hindpaw in cold (10 °C) water and the contralateral hindpaw remained untreated. Mice were pretreated with pharmacological inhibitors or the respective vehicle (i.v., 5 min) before local cold exposure. (**a**) Effects of CGRP receptor antagonist CGRP_8–37_ (400 nmol kg^−1^, *n*=7) on the cold-induced vascular response in WT mice. (**b**) Cold-induced vascular response in WT mice pretreated with a combination of CGRP_8–37_ and NK_1_ receptor antagonist SR140333 (480 nmol kg^−1^, *n*=8) with or without the non-selective NOS inhibitor L-NAME (15 mg kg^−1^, *n*=7) and control (0.01% BSA in saline, *n*=10). (**c**) Effects of CGRP_8–37_, SR140333 and the selective nNOS inhibitor SMTC (10 mg kg^−1^, *n*=6) or control (0.01% BSA in saline, *n*=7) on the cold-induced vascular response in WT mice. All error bars indicate s.e.m. *P<0.05, ***P<0.001, ****P<0.0001 versus respective untreated, ^###^*P*<0.001, ^####^*P*<0.0001 versus cold-treated hindpaw (analysis of variance, Bonferroni *post hoc* test). (**d**) Proposed cold-induced TRPA1 pathway in the regulation of cutaneous vasculature. Local cold exposure (10 °C) causes a transient and rapid decrease in blood flow from baseline (1). This initial phase of cold-induced vasoconstriction consists of activation of TRPA1 which further mediates to (a) the release of noradrenaline (NA) and (b) increased reactive oxygen species release (for example, superoxide (O_2_^−^) generation) that can also activate the ROCK-mediated pathways and increase constriction via phosphorylated MLC-induced increase in [Ca^2+^]_i_. The O_2_^−^ induces the translocation of α_2C_-adrenoceptors from the Golgi to the surface membrane and increases adrenergic activity in the VSMC in a ROCK-dependent fashion^25^. (2) The restoration of blood flow following local cold treatment is essential in restoring blood flow to baseline to protect against local cold-induced injuries. This phase is mediated by the release of CGRP, substance P and nitric oxide following TRPA1 activation of sensory neurons. MLC-P, myosin light chain phosphatase; NA, noradrenaline; p-MLC; phosphorylated myosin light chain; VSMC, vascular smooth muscle cell.

The localization of TRPA1 to sensory neurons expressing the neuropeptide CGRP and substance P led us to investigate the involvement of these vasodilators in the cold-induced vascular response. It is known that TRPA1 agonist can mediate an increase in cutaneous blood flow *in vivo*^12^,^14^. Pretreatment with the CGRP receptor antagonists CGRP_8–37_ or BIBN4096 in WT mice caused a significant loss of the cold-induced restorative response (Figs 4h and 5a). A similar trend was observed in WT mice pretreated with the substance P NK_1_ receptor antagonist SR140333 (Supplementary Fig. 8). Co-treatment with both CGRP_8–37_ and SR140333 in WT mice did not further inhibit the cold-induced vascular responses (Fig. 5b). Whilst a major involvement of the neuropeptides is clear, the data suggest that other mechanisms are also involved. The classic vasodilator prostaglandins are not involved as the cyclooxygenase inhibitor indomethacin had no effect (Supplementary Fig. 9). By comparison, pretreatment of WT mice with the non-isoform selective nitric oxide synthase (NOS) inhibitor L-NAME (L^G^-nitro-L-arginine methyl ester) caused a significant decrease in the response (Supplementary Fig. 10). Moreover, co-treatment with CGRP_8–37_, SR140333 and L-NAME abolished the cold-induced vascular response (Fig. 5b). Whilst a selective endothelial NOS inhibitor is unavailable, pretreatment with the selective neuronal NOS inhibitor, *S*-methyl-L-thiocitrulline (SMTC) alone inhibited the cold-induced vascular response (Supplementary Fig. 11) and in combination with the neuropeptide receptor antagonists, the response was substantially attenuated (Fig. 5c). These data highlight that the combined role of vasodilating sensory neuropeptides and NO mediates the recovery of normal blood flow following the initial constrictor response to local cooling.

## Discussion

This study provides evidence that TRPA1 is an essential vascular sensor of cold, playing a primary role in the vascular response to noxious cooling. Development of a murine model of local cooling, which simulates exposure and recovery to local cold and can be modified by pharmacological and genetic means, provides a new insight into the mechanisms underlying cold-induced vascular responses, first discovered in 1930 (ref. 1). The major novel findings are: (1) TRPA1 acts as a vascular cold sensor to ‘kick start’ the protective cold-induced vascular response as the constrictor response is not observed in the presence of TRPA1 antagonists or in TRPA1 KO mice (2) the initial TRPA1-dependent cold-induced vasoconstriction involves TRPM8 and is mediated via stimulation of α_2C_-adrenoceptors following superoxide generation and involving Rho-kinase-mediated MLC phosphorylation downstream of TRPA1 activation and (3) the TRPA1-mediated cold-induced restorative vasodilator component of the reflex is mediated by the sensory nerve-derived neuropeptides CGRP and substance P, and nNOS-derived NO following TRPA1 activation.

Physiological human studies have described cold pain perception at low temperatures (<15 °C) (ref. 28). In this study, we established a novel acute cold exposure model to enable the quantitative measurement of vascular responses following the local cooling of the hindpaw of anaesthetised mice. Local cold air exposure (28 to 5 °C) has been previously demonstrated to reduce blood flow and skin temperature of mouse hindpaw, with reproducible results derived at 10 °C (ref. 24). Here immediately after local cold water immersion (10 °C for 5 min), a decrease in blood flow was observed, followed by a gradual increase in blood flow to restore blood flow to baseline levels, with minimal reflex changes in the untreated hindpaw. The cold-induced vascular response observed in this study did not involve the previously reported Hunting reaction in cold-induced vasodilatation, which is characterized by cyclic oscillations in blood flow induced by subsequent vasoconstrictions and vasodilatations in extremities measured during the active cooling phase^1^,^3^. We were unable to image blood flow during the actual cooling phase, although we determined a significant vasoconstriction during this phase, through use of a ^99m^Tc clearance technique. It is known that excessive vasoconstriction and/or lack of vasodilatation in response to cold or in Raynaud’s disease causes tissue hypoxia, and in extreme cold conditions, this can result in cyanosis^4^. Thus, there is an urgent need to fully understand the mechanisms involved.

TRPA1 deletion did not change systemic blood pressure and heart rate, highlighting that TRPA1 does not play a role in regulating baseline blood pressure, although TRPA1 may be involved in cardiovascular autonomic reflexes associated with vasovagal reflexes^13^. Furthermore, TRPA1 antagonists have no effects in altering core body temperature in mice^29^ nor does TRPA1 act as a thermoregulator^30^. TRPA1 was initially reported as a cold sensor when Story *et al*.^6^ demonstrated that cold temperatures (<17 °C) induced Ca^2+^ influx in TRPA1 expressing-Chinese Hamster Ovary cells using Ca^2+^ imaging studies. Later, Andersson *et al*.^31^ showed an increase in paw withdrawal latencies in TRPA1 KO mice when placed on a cold plate at 10 °C. The current study was carried out using the same strain of mice. The cold reflex comprising of vasoconstriction followed by restoration of blood flow to baseline levels, (increased blood flow) after exposure of the ipsilateral hind paw to environmental cooling (10 °C for 5 min) was substantially reduced in TRPA1 KO mice and in WT mice pretreated with the TRPA1 antagonist HC030031. The local nature of the response was confirmed by the finding that blood flow remained relatively stable in the untreated contralateral paw, irrespective of treatment. This provided clear evidence that TRPA1 plays a primary role as a vascular cold sensor.

The role of TRPA1 as a noxious cold pain sensor has been a controversial issue. Studies involving TRPA1 KO mice have yielded conflicting results, where (1) Bautista *et al*.^32^ showed no change in cold-induced nociceptive responses (−10 to −20 °C), (2) Kwan *et al*.^33^ demonstrated a decrease in cold (0 °C)-induced pain sensitivity *in vivo* and (3) there were cold-sensing deficits following prolonged cold exposure *in vitro* (13 °C) and in cold (0 °C) plate test *in vivo*^9^. These differences may arise due to the different methodologies used and temperatures studied. However, our present finding that TRPA1 initiates the cold-induced vascular response means that it is possible that changes in blood flow could have influenced the various recordings made and thus contribute to the lack of reproducible findings.

Thermosensitive TRPM8 (<25 °C) is expressed on sensory neurons and mediates the response to deep body cooling which is associated with cutaneous vasoconstriction^18^. TRPM8 is considered to be implicated in thermoregulation as TRPM8 antagonists caused a change in core body temperature in conscious mice and rats^18^. In addition, TRPM8 was shown to override TRPA1 in cold (5–30 °C) detection in neural and behavioral studies *in vivo*^34^, suggesting that TRPM8 rather than TRPA1 is required for acute cold pain sensing in mammals. In our studies, pharmacological blockade or genetic deletion of TRPM8 caused a significant loss of the cold-induced vascular responses, but to a lesser extent than the pharmacological blockade or genetic deletion of TRPA1. Moreover, a TRPM8 antagonist did not further modify the vascular response in TRPA1 KO mice in our model. This finding provides confirmation that TRPA1 is the primary vascular cold sensor in this model, overriding TRPM8 activity and contrasts with evidence that the selective ablation of TRPM8 neurons *in vivo* induced a loss of nociceptor sensitivity to both innocuous (17 °C) and noxious (0 °C) cold^19^.

Our findings illustrate a prominent role of TRPA1 in mediating the cold-induced vascular response, whereas a major involvement of sympathetic nerves in mediating cold-induced vasoconstriction is widely accepted^35^. Indeed, sensory and sympathetic nerves have a close overlapping distribution pattern around blood vessels in the periphery and a reciprocal trophic influence was observed following the denervation of either the sensory peptide- or catecholamine-containing sympathetic nerves^36^. Whilst the pharmacological depletion of sympathetic nerves using guanethidine^24^ or blockade of α-adrenoceptors using phentolamine had a partial inhibitory effect on the total cold-induced vasoconstriction, we observed a decrease in total noradrenaline concentrations in the cold-treated hindpaw at maximum vasoconstriction in TRPA1 WT but not KO mice, compared to untreated samples. This finding is in agreement with previous studies in plasma from human cold (5 °C) studies^37^ and in cutaneous dog veins (37–28 °C) (ref. 38). The observed changes in noradrenaline concentrations in the paw were TRPA1-dependent, implying an involvement of sympathetic nerves.

Superficial cutaneous vessels exhibit increased responsiveness during cooling^35^,^39^ and it is established that α_1_- and α_2_-adrenoceptors mediate peripheral vasoconstriction. All the adrenergic antagonists that we tested exhibited significant effects on the local cold-induced vasoconstriction including the selective α_2_-adrenoceptors antagonist yohimbine, in agreement with previous findings in humans^40^. Different subtypes of α_2_-adrenoceptors are localized in small veins and arteries^41^ and cold treatment has previously been shown to amplify α_2C_- adrenoceptors in isolated mouse tail arteries *in vitro*^23^. We demonstrated that the pharmacological antagonism of α_2C_-adrenoceptors using JP1302 caused a significant decrease in the local cold-induced vasoconstriction.

α_2C_-adrenoceptors are normally silent at thermoneutral temperatures (37 °C), but following cooling (28 °C), they are distributed to the plasma membrane^42^. Although the mechanisms underlying this phenomenon are unclear, both reactive oxygen species and Rho-kinase have been suggested to be involved^25^ and this was directly investigated *in vivo* during this study. We showed that pretreatment with the SOD mimetic TEMPOL caused a significant decrease in cold-induced vasoconstriction in WT mice, highlighting that TEMPOL may reduce the accumulation of superoxide to water and oxygen in the cold-treated hindpaw, consistent with previous *in vitro* findings involving the tail artery^26^. We further measured the levels of superoxide in hindpaw tissue samples at the maximum vasoconstriction phase and provided novel evidence showing that cooling induced an increase in total superoxide levels in the cold-treated hindpaw in a TRPA1-dependent manner. This is the first study to highlight an association between TRPA1 activation and superoxide generation following local cooling treatment *in vivo*. We propose that superoxide is produced downstream of TRPA1 activation by cold exposure in the peripheral vasculature and upstream of α_2C_-adrenoceptor activity, as the α_2C_-adrenoceptor antagonist JP1302 was able to block constriction, but not superoxide production. Of note, mito-TEMPO inhibited the vasoconstriction, providing evidence that the mitochondria are the cellular source.

Superoxide has been suggested to cause vasoconstriction through the Rho-kinase/ROCK-mediated pathways in the vascular smooth muscle cells^25^,^26^. Rho-kinase can also be stimulated by noradrenaline and/or superoxide in response to localized cooling^26^. We investigated the role of the Rho-kinase-mediated pathways in our cold model. WT mice were pretreated with the selective cell-permeable Rho-kinase inhibitor Y27632, which was previously shown to have no modulatory effects on α_2_-adrenoceptors *in vitro* in control experiments (37 °C) (ref. 25). Pretreatment with Y27632 completely abolished the cold-induced vasoconstriction at 10 °C, in agreement with previous *in vitro* cooling (28 °C) studies in mouse tail artery using pressure myography^25^. Hence, our results support previous *ex vivo* findings and provide new evidence demonstrating that cold-induced vasoconstriction at a lower temperature (10 °C) is dependent on the generation of superoxide following activation of TRPA1, which further leads to activation of Rho-kinase and translocation of α_2C_-adrenoceptors to the cell surface membrane for activation in the mouse hindpaws.

Rho-kinase can modulate smooth muscle contraction via an increase in MLC activity^27^. Stimulation of postsynaptic α_2_-adrenoceptors using a selective α_2_-agonist, UK14304 was previously shown to increase vascular tone via an increase in [Ca^2+^]_i_ and MLC phosphorylation in spiral strips of rabbit saphenous vein *in vitro*^43^. We hypothesized that there was an increase in MLC phosphorylation in the local cold-induced vasoconstriction phase. Indeed, we provide evidence demonstrating that there was an increase in phosphorylated MLC protein in cold-treated hindpaw tissue samples at the maximum vasoconstriction phase of TRPA1 WT but not KO mice. Cumulatively, these findings provide a novel mechanism proposing that local cold exposure activates TRPA1, which causes an increase in superoxide generation, activating Rho-kinase to phosphorylate MLC and/or translocate α_2C_-adrenoceptors, ultimately leading to vasoconstriction in the mouse hindpaw.

The role of TRPA1 in mediating vasorelaxant effects is well documented in the literature^12^,^13^,^15^, with little evidence of the vascular constrictor activity. Hence, we further determined if endogenous TRPA1 activation was responsible for initiating the restorative phase that follows the constrictor activity in this cold model. To determine this, we treated WT mice with HC030031 (i.v.) during the vasoconstriction phase following local cold water immersion. The results showed that although the constrictor phase was able to proceed normally following cold exposure, the acute treatment with the TRPA1 antagonist (i.v.) prevented the vasodilator/recovery phase.

TRPA1 is expressed on sensory neurons and cold-induced responses are known to be controlled neurally^1^,^5^,^44^, with conflicting evidence of mechanisms in the literature^3^. Since TRPA1 is co-expressed in TRPV1-positive sensory neurons^6^,^20^, possible interactions between the two channels may occur. We show that neither pharmacological blockade with two TRPV1 antagonists, nor TRPV1 deletion influenced the cold-induced response. TRPV4 has been suggested to act as an environmental cutaneous thermosensor^21^, but TRPV4 deletion did not affect the response, consistent with the knowledge that there was no change in core body temperature between TRPV4 WT and KO mice following a cold stress (4 °C for 150 min)^45^.

On the other hand, the link between TRPA1 and the major sensory neuropeptide dilator CGRP is clear. Our studies using the non-peptide selective antagonist BIBN4096 showed that only the restorative vasodilation phase was affected, which is important in protecting against local cold-induced injury but not the initial constriction component. The pharmacological blockade of either CGRP receptors or the substance P NK_1_ receptors caused a significant reduction in the cold-induced restorative response, indicating a key involvement of sensory nerves in this model. A finding was supported here by sensory nerve depletion studies with resiniferatoxin. Whilst we gained no evidence for a role for vasodilator prostaglandins, pretreatment with L-NAME significantly reduced the cold-induced residual vasodilator response, as was observed in previous cooling (4 °C) studies in human skin^46^. A selective endothelial NOS isoform inhibitor is unavailable, but we show that nNOS-derived NO is involved in mediating the restorative vasodilator response following local skin cold treatment at 10 °C, using the selective nNOS inhibitor SMTC^47^. This is the first study establishing a link between nNOS-derived NO and TRPA1 in mediating vasodilator responses. The pretreatment of mice with a combination of the neuropeptide receptor antagonists and SMTC or L-NAME was able to substantially block the cold-induced blood flow responses, with no significant change in the untreated hindpaw. Thus, there is a clear involvement of the vasodilator sensory neuropeptides and NO in the cold-induced restorative component. The importance of sensory neurogenic vasodilator responses is unclear; here we provide evidence for their functional importance in restoring cutaneous baseline blood flow to its healthy level, rather than acting in a capacity that leads to obvious increased net blood flow over baseline. This is indicative of a physiologically important role, allowing the restoration of a healthy skin flow, necessary to protect the skin against cold-induced injury. CGRP has been shown to tonically cross-inhibit cold-sensitive spinal neurons^48^. We did not observe this in terms of blood flow, just that the normal ability to return to basal blood flow was lost in the absence of functioning neurogenic vasodilation. Whilst the stimulation of sensory nerves with either exogenous TRPA1 or TRPV1 agonists is known to lead to neurogenic vasodilation, the importance of this TRPA1-mediated role of sensory neurogenic vasodilatation in restoring a healthy baseline blood flow to skin has not been defined before. This should be considered as distinct from the increased blood flow commonly observed in response to stimulation of sensory nerves by TRPV1 (for example, flushing in response to the TRPV1 agonist capsaicin).

Collectively, this novel evidence highlights the important role of TRPA1 in (1) sensing the change in temperature at 10 °C, (2) inducing the initial vasoconstriction phase and (3) stimulating the release of sensory nerve-derived neuropeptides and NO, which are essential in replenishing the blood flow that acts to protect against local cold-induced injury (Fig. 5d). To conclude, we have revealed a primary role of the TRPA1 in orchestrating the vascular response to local cold exposure. This finding is consistent with TRPA1 playing a primary physiological role, as a vascular sensor for noxious cold and that as a consequence is also important for the pathophysiological understanding of peripheral cold injury and disease. The results introduce a new paradigm whereby TRPA1 is an important activation step, acting in two distinct ways to affect the successful vascular protective response to local noxious cold exposure.

## Methods

### Animals

Male mice (8–12 weeks of age) were used in all experiments. The initial characterization of cold-induced vascular responses was performed in CD1 mice, purchased from Charles River (Kent, UK). TRPA1 KO mice (C57BL/6-B6129P1/F2J mixed genetic background) and WT littermates were bred from heterozygotic mice kindly provided by Drs Kelvin Kwan (Harvard Medical School, Boston, MA) and David Corey (Harvard Medical School, Boston, MA)^33^. TRPM8 KO^49^, TRPV1 KO^50^ and TRPV4 KO^45^ were raised on a C57/BL6 background and their WT littermates were used as controls. All genetically modified mice were backcrossed for at least eight generations into their respective background. Mice were housed in a climatically controlled environment, on a 12-h light/dark cycle, with free access to water and standard food *ad libitum*. All experiments were conducted in accordance with the UK Home Office Animals (Scientific Procedures) Act, 1986 and were approved by the King’s College London Animal Care and Ethics Committee. All experiments were conducted in a blinded manner. Animals were randomly assigned to control or treatment groups and the experimenter was blinded towards the genetic background of animals at the time of experiment.

### Cutaneous blood flow by full-field laser perfusion imager

Mice were anaesthetized i.p. with ketamine (75 mg kg^−1^) and medetomidine (1 mg kg^−1^) and cutaneous blood flow was assessed in the whole plantar hindpaw area using the Full-Field Laser Perfusion Imager (FLPI, Moor Instruments, UK)^12^,^14^. The environmental cooling model consisted of recording baseline blood flow in both hindpaws to ensure the haemodynamic vascular responses have stabilized following anaesthesia. The ipsilateral hindpaw was subsequently exposed up to the level of the joint between the tibia and the calcaneum to cold water (10 °C for 5 min). The contralateral paw was untreated at room temperature (19–21 °C) and served as a control. Changes in blood flow after these treatments were followed for 30 min. Mice were placed on a heating mat, in the ventral position maintained at 36 °C for blood flow measurements. The response to cooling consisted of an initial reduced flux, in keeping with vasoconstriction, followed by a slow developing sustained increased flux, consistent with vasodilatation. Water is a much more efficient medium at cooling down tissues and this model allowed blood flow responses to be monitored following the local cooling treatment. Results are expressed as (1) a measure of maximum % decrease in blood flow from baseline to 0–2 min following cold treatment or (2) arbitrary flux units ( × 10^3^ flux units) measured as area under the recorded flux (response curve) versus time for the entire recording period for 30 min following cold treatment (Supplementary Fig. 1).

### Cutaneous blood flow in the ear by laser Doppler techniques

Skin blood flow was measured concomitantly in both ears of mice anaesthetized with ketamine (75 mg kg^−1^) and medetomidine (1 mg kg^−1^) using laser Doppler flowmeter (Moor instruments, UK) and a PowerLab data acquisition system (AD instruments, UK) or Full-Field Laser Perfusion Imager (FLPI)^13^. A probe, allowing blood flow to be measured precisely at one point in the ear (1 mm^2^ and to 1–2 mm depth), was placed at one point of each ear and a baseline reading was obtained for 2–5 min. Cinnamaldehyde (20 μl, 10%) or capsaicin (200 μg solution) was topically applied to the ipsilateral ear and vehicle; 10% dimethylsulphoxide (DMSO) in ethanol or 100% ethanol, respectively, on the contralateral ear. Blood flow was subsequently measured for 30 min (cinnamaldehyde studies) or 20 min (capsaicin studies), and data collected as flux units that are proportional to blood flow. All experiments using cinnamaldehyde administration was conducted using the laser Doppler flowmeter and in some experiments, images were captured using FLPI, whilst all experiments using capsaicin were conducted using the FLPI. The typical response to cinnamaldehyde or capsaicin involves an initial gradual increase in blood flow from baseline, as a result of vasodilatation. Blood flow data were expressed as area under the recorded flux versus time trace for the entire recording period (30 min) following topical treatment.

### Changes in blood flow by ^99m^Tc clearance technique

Blood flow was assessed using a clearance technique in paw tissues^51^. Test agents containing an equal amount of radioactive ^99m^technetium (^99m^Tc) were prepared immediately before use. Mice were pretreated with control (10% DMSO in saline, i.p., 30 min) or TRPA1 antagonist HC030031 (100 mg kg^−1^, i.p., 30 min), anaesthetized with ketamine (75 mg kg^−1^) and medetomidine (1 mg kg^−1^) and saline was co-injected with an equal amount of radioactive ^99m^Tc (20kBq per site, intradermally (i.d.) 30 μl; Nuclear Medicine, Guy’s Hospital, London) in the plantar skin of WT mice. An equal amount of saline+^99m^Tc was used as a measurement of total radioactivity. Following i.d. injection, the ipsilateral hindpaw was immersed in local cold water (10 °C) for 5 min and the contralateral paw remained untreated. At 0–2 min following local cold exposure, the clearance period was terminated by cervical dislocation and death, which reduce blood flow to zero. The paw tissues were rapidly collected and the remaining radioactivity was immediately counted in an adjacent radioactive gamma counter. Clearance was calculated by comparison of the paw tissue and total radioactivity readings. Local cold treatment-specific clearance in the ipsilateral paw was then calculated by comparison with the untreated contralateral paw, normalized to 100% and expressed as a % change in clearance compared to the untreated paw. Positive values are due to decreased clearance, which is directly related to decrease blood flow and vasoconstrictor activity.

### Radiotelemetry

Blood pressure and heart rate were measured using a radiotelemetry device (PA-C10, DSI, NL), with the catheter placed in the left carotid artery and advanced towards the aortic arch^52^. It was secured using surgical braided silk (5.0, waxed, Pearsalls sutures) and the outer wound was closed with absorbable sutures (4.0, Ethicon, Johnson and Johnson) in a discontinuous pattern. The transmitter was placed subcutaneously in the left flank and the transmitter pocket was irrigated with sterile saline. All procedures were conducted using aseptic techniques under isoflurane anaesthesia (2.5%, Abbott Laboratories, UK) in 3 l min^−1^ O_2_. Surgical anaesthesia was assessed by loss of the paw pinch reflex. Buprenorphine was administered intramuscularly (50 μg kg^−1^, Vetergesic, Alstoe Animal Health) for pain relief. All animals were housed singly and allowed to recover for 7 days before recording baseline blood pressure for three continuous days in a quiet room. Data were collected and calculated by the DSI software (DSI Dataquest A.R.T.) and analysed in Microsoft Excel and GraphPad Prism 5.

### Antagonists and inhibitors

The TRPA1 antagonist HC030031 (2-(1,3-dimethyl-2,6-dioxo-1,2,3,6-tetrahydro-purin-7-yl)-*N*-(4-isopropyl-phenyl)-acetamide 1) (Tocris, UK) was either dissolved in 10% DMSO in saline and administered i.p. at a dose of 100 mg kg^−1^ 30 min^53^ before local cold water immersion or in 8% DMSO, 2% Tween-80 in saline and administered i.v. at a dose of 6 mg kg^−1^ (ref. 54) at the peak vasoconstriction phase (0–2 min) following local cold water immersion. The TRPM8 antagonist AMTB (*N*-(3-aminopropyl)-2-[(3-methylphenyl) methyl]oxy-*N*-(2-thienylmethyl)benzamide hydrochloride salt) was dissolved in 10% DMSO in saline, TRPV1 antagonist SB366791 (4′-Chloro-3-methoxycinnamanilide) in 2% DMSO in saline or AMG9810 ((2*E*)-*N*-(2,3-Dihydro-1,4-benzodioxin-6-yl)-3-[4-(1,1-dimethylethyl)phenyl]-2-propenamide) (Sigma, UK) in 2% DMSO and 5% Tween-80 in saline and administered at a dose of 10 (ref. 55), 25 (ref. 56) and 50 mg kg^−1^ (ref. 57), respectively. The CGRP receptor antagonists were dissolved as follows: CGRP_8–37_ was dissolved in 0.01% bovine serum albumin (BSA) and administered i.v. at 400 nmol kg^−1^ (ref. 58) (Tocris,UK). BIBN4096 ((1-piperidinecarboxamide,*N*-[2-[[5-amino-1-[[4-(4-pyridinyl)-1-piperazinyl]carbonyl]pentyl]amino]-1-[3,5-dibromo-4-hydroxyphenyl)methyl]-2-oxoethyl]-4-(1,4-dihydro-2-oxo-3(2H)-quinazolinyl)-,[R-(R*,S*)]) (Tocris, UK) was dissolved in a minimum volume of 1M HCl (0.5 mg per 50 μl) and the solution was made up to the required final volume with saline at a final stock concentration of 2 mg ml^−1^, and then titrated with 1 M NaOH to return the pH to neutral. BIBN4096 was administered i.v. at 0.3 mg kg^−1^ (refs 59, 60). The NK_1_ receptor antagonist SR140333 ((*S*)1-(2-[3-(3,4-dichlorophnyl)-1-(3-isopropoxyphenylacetyl)piperidin-3-yl]ethyl)-4-phenyl-1-azoniabicyclo[2.2.2]octane chloride)^60^, a gift from Dr X. Emonds-Alt, Sanofi, Toulouse, France, was dissolved in saline and administered at a dose of 480 nmol kg^−1^ i.v. The non-selective NOS inhibitor L-NAME^60^ or the selective nNOS inhibitor SMTC (Sigma, UK) was dissolved in saline and administered i.v. at 15 mg kg^−1^ (ref. 61). The cyclooxygenase inhibitor indomethacin (Sigma, UK) was dissolved in 5% NaHCO_3_ in saline and administered i.v. at 20 mg kg^−1^ (ref. 60). The non-selective α-adrenoceptor antagonist phentolamine^62^ was dissolved in saline and administered i.v. at a dose of 10 mg kg^−1^, whilst the sympathetic nerve blocker guanethidine^63^ (Sigma, UK) was dissolved in saline and administered subcutaneously (s.c.), at a dose of 30 mg kg^−1^ daily for four consecutive days. The α_2_-adrenoceptor antagonist yohimbine hydrochloride, the selective α_2C_-adrenoceptor antagonist JP1302 dihydrochloride and the selective Rho-associated protein kinase (ROCK) inhibitor Y27632 (Tocris, UK) were dissolved in saline and administered at a dose of 10 mg kg^−1^ (i.p.)^64^, 3 μg kg^−1^ (s.c.)^65^ or 5 mg kg^−1^ (i.p.)^66^, respectively. The SOD mimetic TEMPOL was dissolved in saline and administered i.v. at a dose of 30 mg kg^−1^ (ref. 60), whilst the mitochondria-targeted superoxide scavenger mito-TEMPO was dissolved in saline and administered i.p. at a dose of 10 mg kg^−1^ (ref. 67). The capsaicin analogue resiniferatoxin was dissolved in 10% ethanol, 10% Tween-80 in saline and administered s.c. at a dose of 0.3 mg kg^−1^ for three consecutive days^68^. These drugs were administered i.v. 5 min, i.p. 30 min and s.c. 60 min before baseline blood flow recording. The doses used were based on previous and preliminary studies.

### Superoxide measurements

Superoxide release from fresh plantar hindpaw skin samples was measured by chemiluminescence using lucigenin (bis-*N*-methylacridinium nitrate; Sigma Aldrich) as a probe. Chemiluminescense was measured using a GloMax 20/20 luminometer (Promega, UK). Skin samples were collected 0–2 min post cold water immersion, which corresponds to the peak vasoconstriction phase of the cold-induced vascular response, as determined by laser speckle imaging studies. The samples were placed in 100 μl of modified Krebs’ buffer (composition of 131 mM NaCl, 5.6 mM KCl, 25 mM NaHCO_3_, 1 mM NaH_2_PO_4_·H_2_O, 5 mM glucose, 5 mM HEPES, 100 μM L-arginine, 2.5 mM CaCl_2_, 1 mM MgCl_2_ and 100 μM NADPH, pH7.4) and a further 100 μl of modified Krebs’ buffer containing lucigenin (10 mM) and NADPH (500 μM) was added to each sample with or without SOD (50 U ml^−1^). Chemiluminescense was recorded after 4 min. Results are expressed as the difference in the relative light units per mg of protein in the presence and absence of SOD after subtraction of background luminescence^69^.

### Western blotting

Plantar hindpaw skin tissues of TRPA1 WT and KO mice were collected 0–2 min post cold water immersion and snap frozen at −80 °C until processing. Tissue was then lysed using in SDS lysis buffer containing protease (one tablet per 50 ml, Roche, Germany) and phosphatase (one tablet per ml, Roche, Germany) inhibitor. Lysates were clarified by centrifuging at 2,600 *g* for 10 min at 4 °C. Protein concentration was assessed using the Bradford dye-binding method kit (Bio-rad). Protein (30 μg) was loaded and separated by SDS–polyacrylamide gel electrophoresis and transferred to polyvinylidene difluoride membranes using a semi-dry technique. Membranes were blocked with 5% BSA in Tris-buffered saline (TBS) and 0.1% Tween and incubated with primary antibodies against MLC-2 (t-MLC, 1:500 dilution, Cell Signaling, UK, #3672) and phospho-MLC (Ser19, 1:500 dilution, Cell Signaling, UK, #3671) in 5% BSA in TBS and 0.1% Tween for 16 h at 4 °C. Membranes were washed further with TBS 0.1% Tween and incubated with a horseradish peroxidase conjugated anti-rabbit secondary antibody (1:2,000 dilution, Sigma, UK, #AP132P). Proteins were detected by enhanced chemiluminescence (ECL, Piercenet, UK) and developed using the Syngene gel doc dark room system (Chicago, USA). Densitometric analysis performed using Image J analysis software (NIH, USA). MLC phosphorylation was assessed by calculating the ratio of anti-phosphorylated-MLC signal to anti-total MLC signal with defined regions, normalized to the loading control β-actin (1:2,000 dilution, Sigma, UK, #A1978). Uncropped images of immunoblots are shown in Supplementary Fig. 12.

### Quantification of noradrenaline using ELISA

Noradrenaline levels were measured using a commercially available Noradrenaline Enzyme Linked Immuno Sorbent Assay (ELISA) kit (RE59261; IBL International, Hamburg, Germany) in hindpaw tissue homogenates of WT and TRPA1 KO mice following cold treatment at 0–2 min. Samples were collected in a sterile environment and snapfrozen at −80 °C until processing. Tissue samples were homogenized in RIPA lysis buffer (Sigma, UK) containing protease inhibitors (one tablet per 50 ml, Roche, Germany) and lysates were clarified by centrifuging at 2,600 *g* for 10 min at 4 °C. According to the manufacturer’s instructions, 20 μl of tissue lysates, standards and controls were added to extraction plates. After extraction, bound noradrenaline (25 μl) was eluted using release buffer and transferred to a 96-well ELISA plate. Noradrenaline antiserum (50 μl) was incubated with all samples at room temperature for 120 min on an orbital shaker; the plates were thoroughly washed with diluted washing buffer and 100 μl of enzyme conjugate was added into each well and incubated for 60 min at room temperature. Following several washes, 200 μl of pNPP substrate solution was incubated at room temperature for 40 min and the reactions were stopped by addition of 50 μl of pNPP stop solution per well. The optical density of each well was measured at 405 nm and a standard curve was plotted using a range of known noradrenaline concentrations provided in the kit (0, 5, 15, 50, 150, 500 ng ml^−1^). Positive and negative controls were included to determine accuracy. The limit of sensitivity was 20 pg ml^−1^ and the linearity limit was 8.0 ng ml^−1^. Cross-reactivity to other catecholamines or metabolites was manufacturer tested as <0.02%. Protein concentrations of each sample was determined using the Bradford dye-binding method (Bio-rad) and noradrenaline concentrations of each sample was normalized to mg of protein and expressed as noradrenaline (ng mg^−1^ tissue protein)^70^.

### Statistical analysis

Most experiments in this study involved four groups and hence, a power analysis for a two-way analysis of variance design was used, based on previously published data from the mouse ear model and hindpaw vasculature 12,13. For confidence at the 0.05 (5%) with power at 0.8 (80%) and the effect size of medium (0.75), groups of *n*=8 are recommended. In some experiments where a difference was clearly distinct, the experiment was unblinded for analysis (sample size >4) to minimize animal use.

Results are expressed as mean±s.e.m. Data statistical analysis was performed using a two-tailed Student’s *t*-test or two-way analysis of variance followed by Bonferroni’s comparison *post hoc* test. *P*<0.05 was considered to represent a significant difference.

## Author contributions

A.A.A. and R.G. characterized the model, performed and analysed data and wrote the manuscript; X.K., K.M.A., J.V.B., S.S., C.G., R.H. and E.S.F. performed the experiments and data analysis; A.D.G. and S.J.B. provided the reagents and advice on analysis. S.D.B. coordinated the project, drafted and wrote the article. All authors contributed to the final version.

## Additional information

**How to cite this article:** Aubdool, A. A. *et al*. TRPA1 is essential for the vascular response to environmental cold exposure. *Nat. Commun.* 5:5732 doi: 10.1038/ncomms6732 (2014).

## Supplementary Material

Supplementary Information Supplementary Figures 1-12, Supplementary Tables 1-2 and Supplementary Reference.
