# Supplementary material for: TRPA1 is essential for the vascular response to environmental cold exposure
Source: Nat Commun. 2014 Dec 11;5:5732. doi: 10.1038/ncomms6732 (PMC4284811; doi:10.1038/ncomms6732)
Supplement: Supplementary Information — Supplementary Figures 1-12, Supplementary Tables 1-2 and Supplementary Reference. [file ncomms6732-s1.pdf]

Supplementary Information

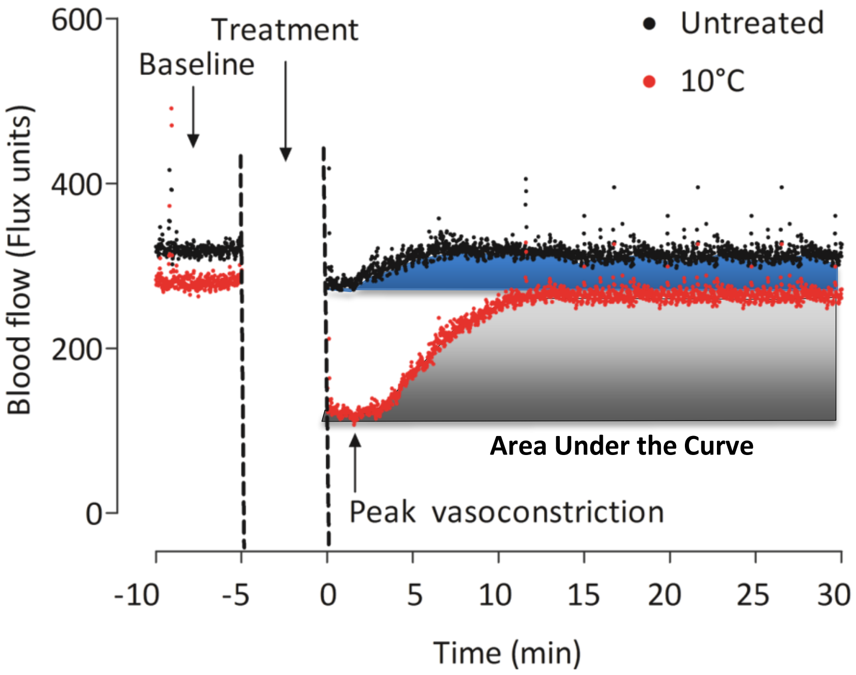

Supplementary Figure 1: Representative trace of blood flow responses analysis. Blood flow was measured by a laser speckle dynamic blood flow perfusion imager. Baseline blood flow was measured for 5 min, the ipsilateral paw was immersed in cold water for 5 min, and the contralateral paw remained untreated. Blood flow was subsequently measured for 30 min post cooling. Parameters derived from this blood flow profile response are (1) Baseline, mean blood flow flux value during the 5 min recording before local cold water immersion, (2) Maximum vasoconstriction, the flux value which is usually at 0-2 min following local cold exposure representing maximum vasoconstriction from baseline following local cold treatment. The area under the curve (AUC) represents restoration of blood flow in the paw for 30 min following cold water immersion (blue for untreated paw and grey for cold-treated paw), expressed as blood flow (x10^3 flux units). The following mathematical calculation was used to drive maximum vasoconstriction.

$$Maximum\ vasoconstriction = \frac{(Peak\ vasoconstriction - Baseline)}{Baseline} \times 100\%$$

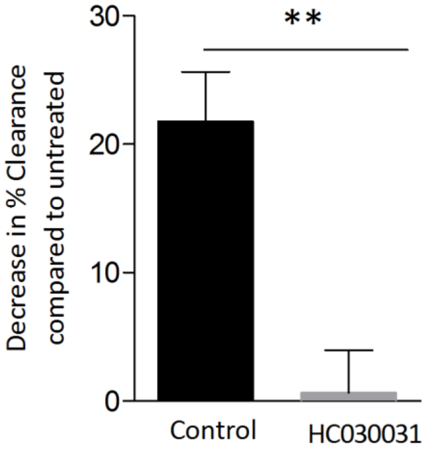

Supplementary Figure 2: Assessment of blood flow during local cold exposure by ^{99m}Technitium (^{99m}Tc clearance) Mice were pre-treated with TRPA1 antagonist HC030031 (100mg kg^{-1}, *i.p.*, 30 min, n=5) or control (10% DMSO in saline, *i.p.*, 30 min, n=6), anaesthetised, injected intradermally via the plantar skin with saline containing an equal amount of ^{99m}Tc (approx. 20kBq per site). The ipsilateral paw was immersed in cold water (10°C for 5 min) and the contralateral paw remained untreated. The experiment was immediately terminated and paws removed for radioactive counting and counts compared with samples of total counts. The results are shown as decrease in % clearance compared with the untreated paw. All error bars indicate s.e.m. **$p<0.01$ versus cold-treated hindpaw in control group (two-tailed Student's t-test).

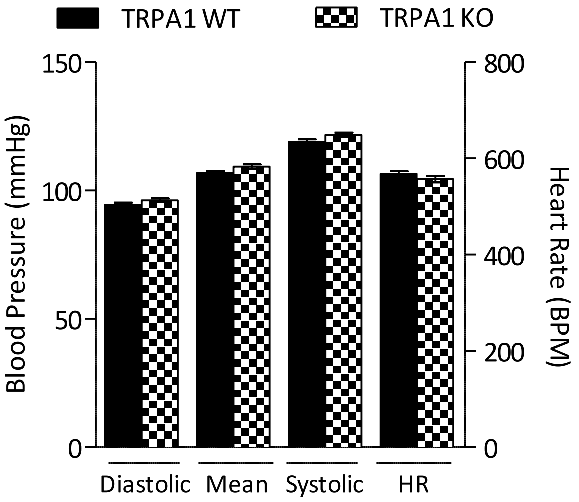

Supplementary Figure 3: Cardiovascular haemodynamics of TRPA1 WT and KO mice. Average diastolic, mean and systolic blood pressure (mmHg) , and heart rate (bpm) were measured in conscious TRPA1 WT and KO mice (n=7-10) on days 10-13 (baseline measurements) following implantation of a radiotelemetry transmitter.

A

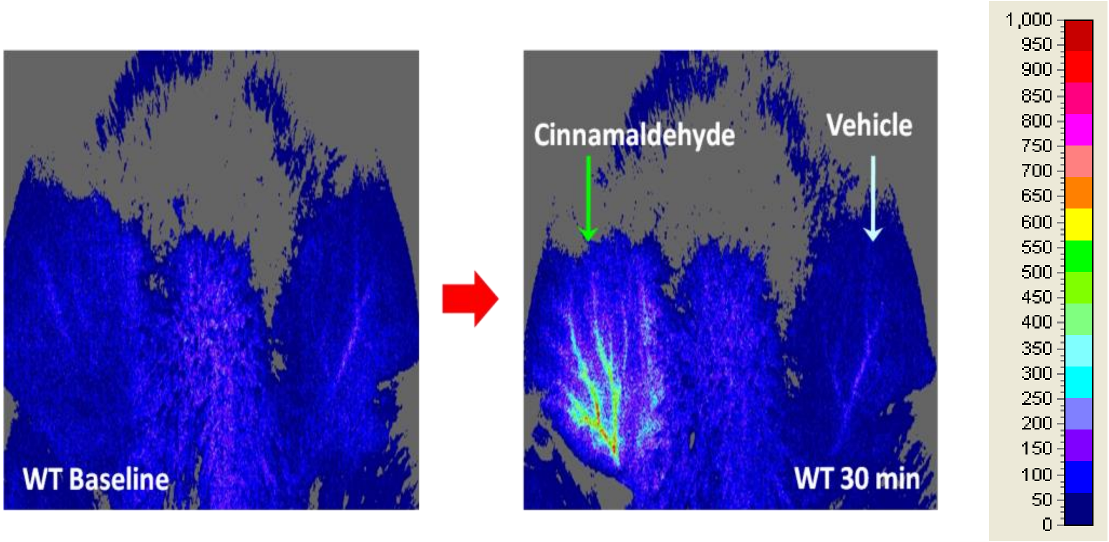

B

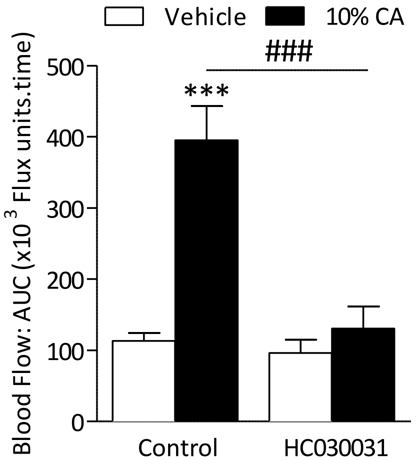

C

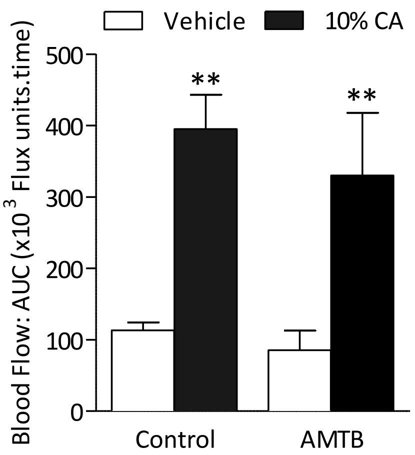

Supplementary Figure 4: Effect of TRPA1 and TRPM8 in cinnamaldehyde-induced vasodilatation. Blood flow (x 10^3 flux units.time) in mouse ears following topical application of cinnamaldehyde (CA, 10%) or vehicle (10% DMSO in ethanol) using laser Doppler techniques. Results recorded over a 30 min period and shown as mean + S.E.M. **A,** Representative images as observed by the Full-Field Laser Perfusion imager (FLPI) where blood flow was recorded at baseline and 30 min post application in WT mouse. Cinnamaldehyde-induced increased blood flow, indicated in the pseudo-colour images in WT mice pre-treated with **B,** the TRPA1 antagonist HC030031 (100mg kg^{-1}, *i.p.*, 30 min) or control (10% DMSO in saline) in male TRPA1 WT mice (n=4-5) and **C,** the TRPM8 antagonist AMTB (10mg kg^{-1}, *i.p.*, 30 min) or control (10% DMSO in saline, *i.p.*, 30 min) (n=5) using laser Doppler flowmeter. All error bars indicate s.e.m. **p<0.01, ***p<0.001, compared to respective vehicle-treated, ###p<0.001 compared to CA-treated ears of WT mice (ANOVA, Bonferroni *post hoc* test).

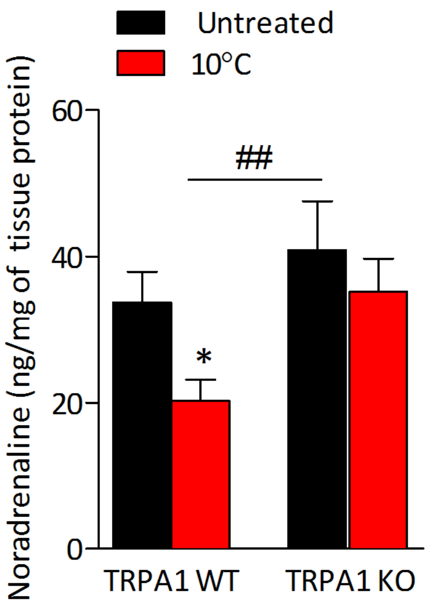

Supplementary Figure 5: Effects of cold treatment on total noradrenaline concentration in TRPA1 WT and KO mice. Noradrenaline concentrations as quantified by ELISA in the hindpaw of TRPA1 WT and KO hindpaw tissue collected at 0-2 min (vasoconstriction phase, n=8-12) following local cold (10°C for 5 min) water immersion. Values are expressed as noradrenaline concentration (ng) per mg of tissue protein. All error bars indicate s.e.m. *$p<0.05$ compared to respective untreated, ##$p<0.01$ compared to cold-treated hindpaws of TRPA1 KO mice (ANOVA, Bonferroni *post hoc* test).

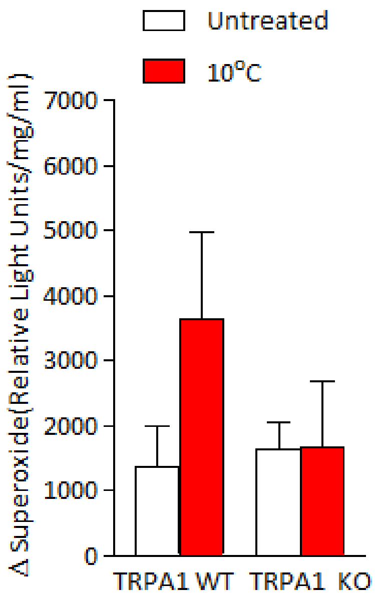

Supplementary Figure 6: Effects of local cold treatment on superoxide generation. Total superoxide levels were measured by Lucigenin chemiluminescence in the hindpaw of TRPA1 WT and KO, in hindpaw tissues collected at 0-2 min (vasoconstriction phase) following local cold (10°C) water immersion. Values are expressed as relative light units per mg of tissue homogenate (n=4-5). All error bars indicate s.e.m. ns, p>0.05 compared to respective untreated, p>0.05 compared to cold-treated hindpaws of TRPA1 KO mice (ANOVA, Bonferroni *post hoc* test).

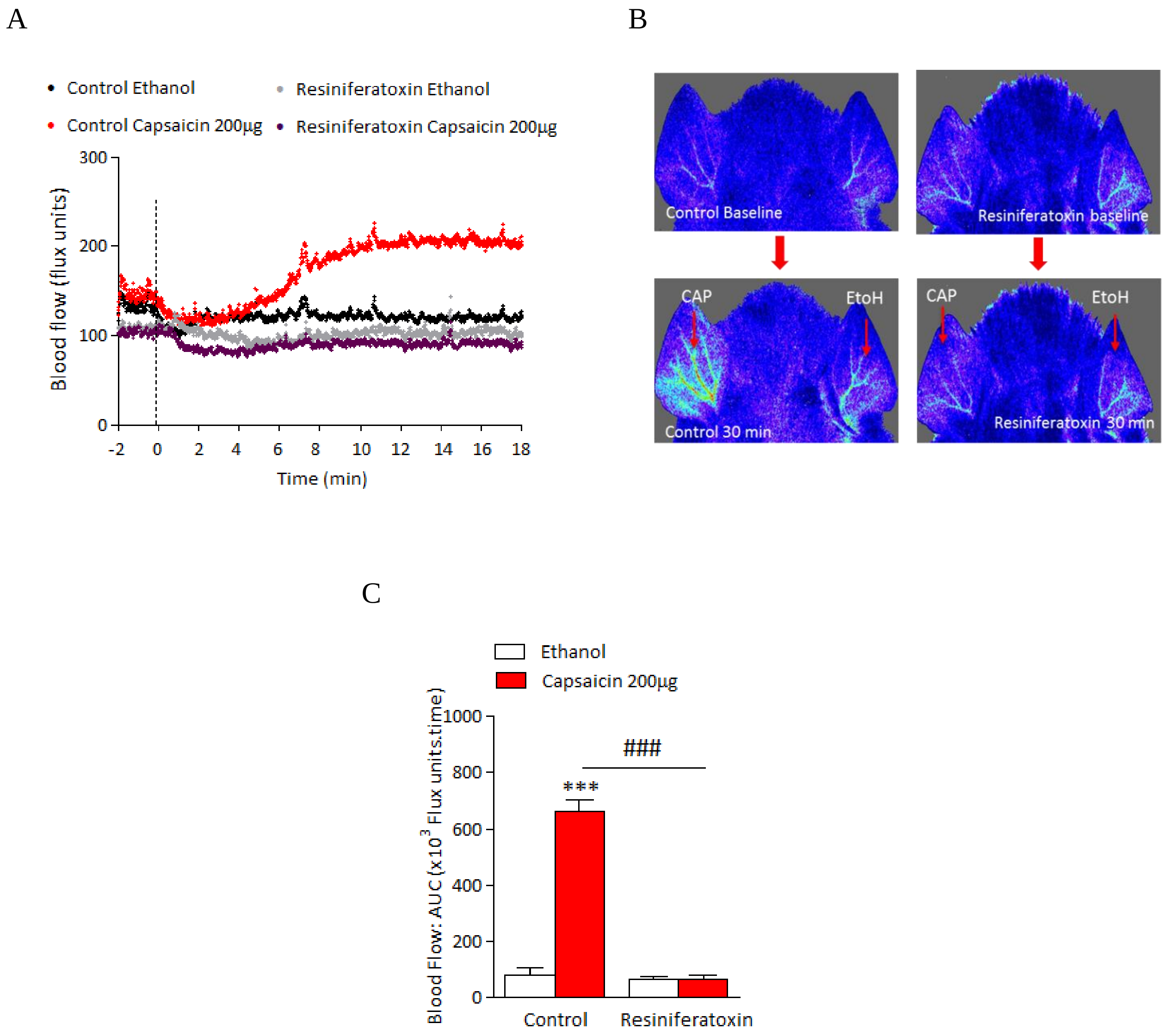

Supplementary Figure 7. Characterisation of sensory nerve denervation using ultra-potent capsaicin analogue resiniferatoxin. WT mice were treated with resiniferatoxin (0.3mg kg^{-1}, *s.c.*, daily, n=13) or control (10% ethanol in 8% Tween-80 in saline, *s.c.*, daily, n=9[1]) for 3 consecutive days. Denervation of sensory neurons was confirmed by investigating blood flow responses to capsaicin at Day 4. Blood flow (x 10^3 flux units) was assessed in the mouse ear following topical application of capsaicin (CAP, 200µg) or vehicle (ethanol) using the Full-field Laser Perfusion Imager (FLPI). Results recorded over a 20 min period and shown as mean + S.E.M. **A,** Representative blood flow trace of capsaicin-induced vasodilatation in WT mice pre-treated with resiniferatoxin or control. Dotted line represents time of topical application of vehicle or capsaicin following baseline measurements. **C,** Representative images as observed by the Full-Field Laser Perfusion imager (FLPI) where blood flow was recorded at baseline and 30 min post application of capsaicin in the ears of WT mice pre-treated with resiniferatoxin or control. **D,** Capsaicin-induced increased blood flow in WT mice with resiniferatoxin (n=10) or control (n=6). All error bars indicate s.e.m ***p<0.001 versus respective ethanol-treated ears, ###p<0.001 versus control-treated groups (ANOVA, Bonferroni *post hoc* test).

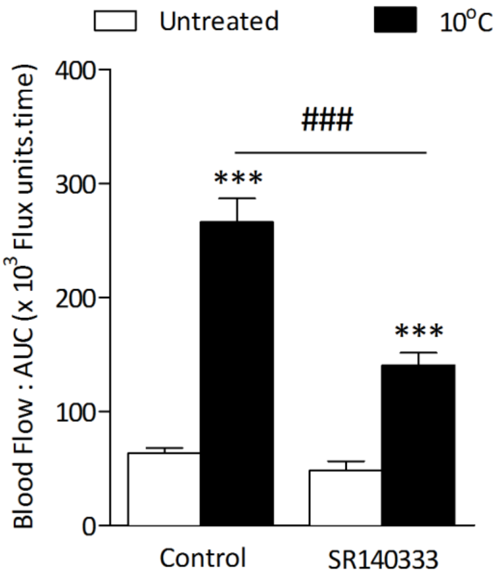

Supplementary Figure 8: Effect of substance P receptor antagonist in cold-induced vascular responses. The blood flow response was measured in WT mice following local immersion of the hindpaw in cold water (10°C) in WT mice pre-treated with the substance P receptor antagonist SR140333 (480nmol kg^{-1}, *i.v.*, 5 min) or control (saline, *i.v.*, 5 min) in WT mice (n=5) using FLPI. Data represents the total cold-induced restorative response (30 min), as assessed as area under the curve. All error bars indicate s.e.m. ***p<0.001 versus respective untreated, ###p<0.001 versus cold-treated hindpaw (ANOVA, Bonferroni *post hoc* test).

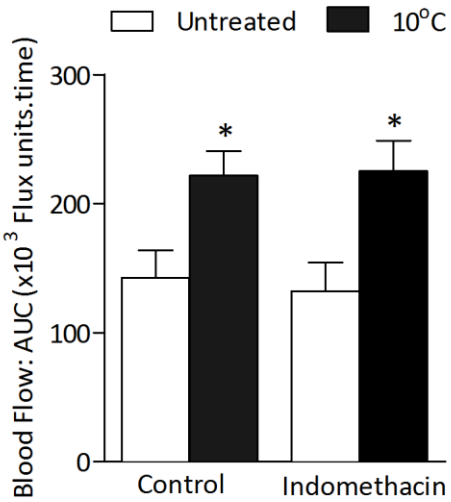

Supplementary Figure 9: Effect of a cyclo-oxygenase inhibitor on the cold-induced vascular response. Blood flow responses were measured in WT mice following local immersion of the hindpaw in cold water (10°C) in WT mice pre-treated with the non-selective cyclo-oxygenase inhibitor indomethacin (5mg kg^{-1}, *s.c.*, 60 min, n=10) or control (5% $NaHCO_3$ in saline, *s.c.*, 60 min, n=12) in WT mice using FLPI. Data represents the total cold-induced restorative response (30 min), as assessed as area under the curve. All error bars indicate s.e.m *$p<0.05$ versus respective untreated hindpaw (ANOVA, Bonferroni *post hoc* test).

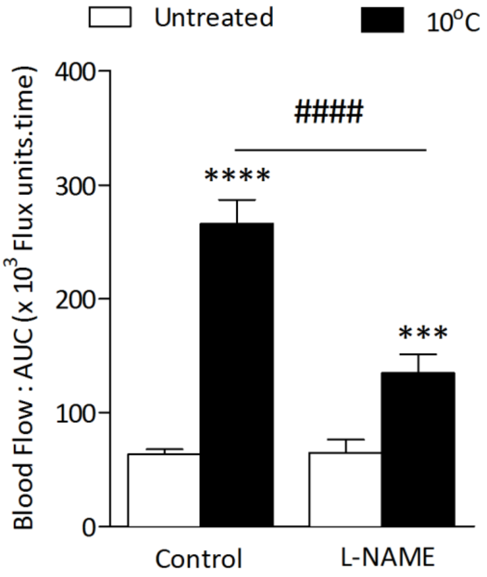

Supplementary Figure 10: Effect of a non-selective nitric oxide synthase inhibitor in the cold-induced vascular response. Blood flow responses were measured in WT mice following local immersion of the hindpaw in cold water (10°C) in WT mice pre-treated with the non-selective nitric oxide synthase (NOS) inhibitor L-NAME (15mg kg^{-1}, *i.v.*, 5 min, n=7) or control (saline, *i.v.*, 5 min, n=10) in WT mice using FLPI. Data represents the total cold-induced restorative response (30 min), as assessed as area under the curve. All error bars indicate s.e.m ***p<0.001, ****p<0.0001 versus respective untreated, ####p<0.0001 versus cold-treated hindpaw (ANOVA, Bonferroni *post hoc* test).

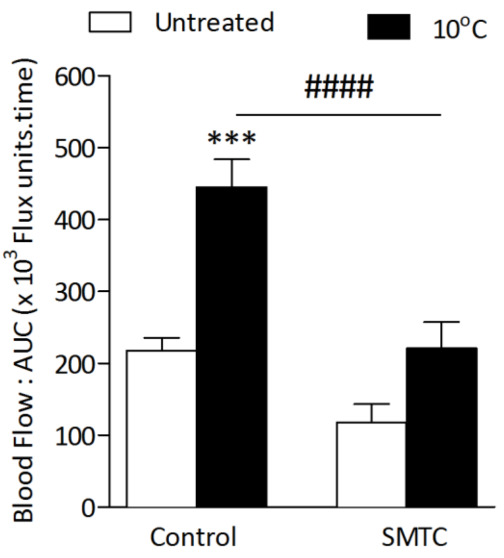

Supplementary Figure 11: Effect of a selective neuronal nitric oxide synthase inhibitor in the cold-induced vascular response. Blood flow responses were measured in WT mice following local immersion of the hindpaw in cold water (10°C) in WT mice pre-treated with the selective neuronal nitric oxide synthase (nNOS) inhibitor SMTC (10mg kg^{-1}, *i.v.*, 5 min, n=6) or control (saline, *i.v.*, 5 min, n=7) in WT mice using FLPI. Data represents the total cold-induced restorative response (30 min), as assessed as area under the curve. All error bars indicate s.e.m ***p<0.001 versus respective untreated, ####p<0.0001 versus cold-treated hindpaw (ANOVA, Bonferroni *post hoc* test).

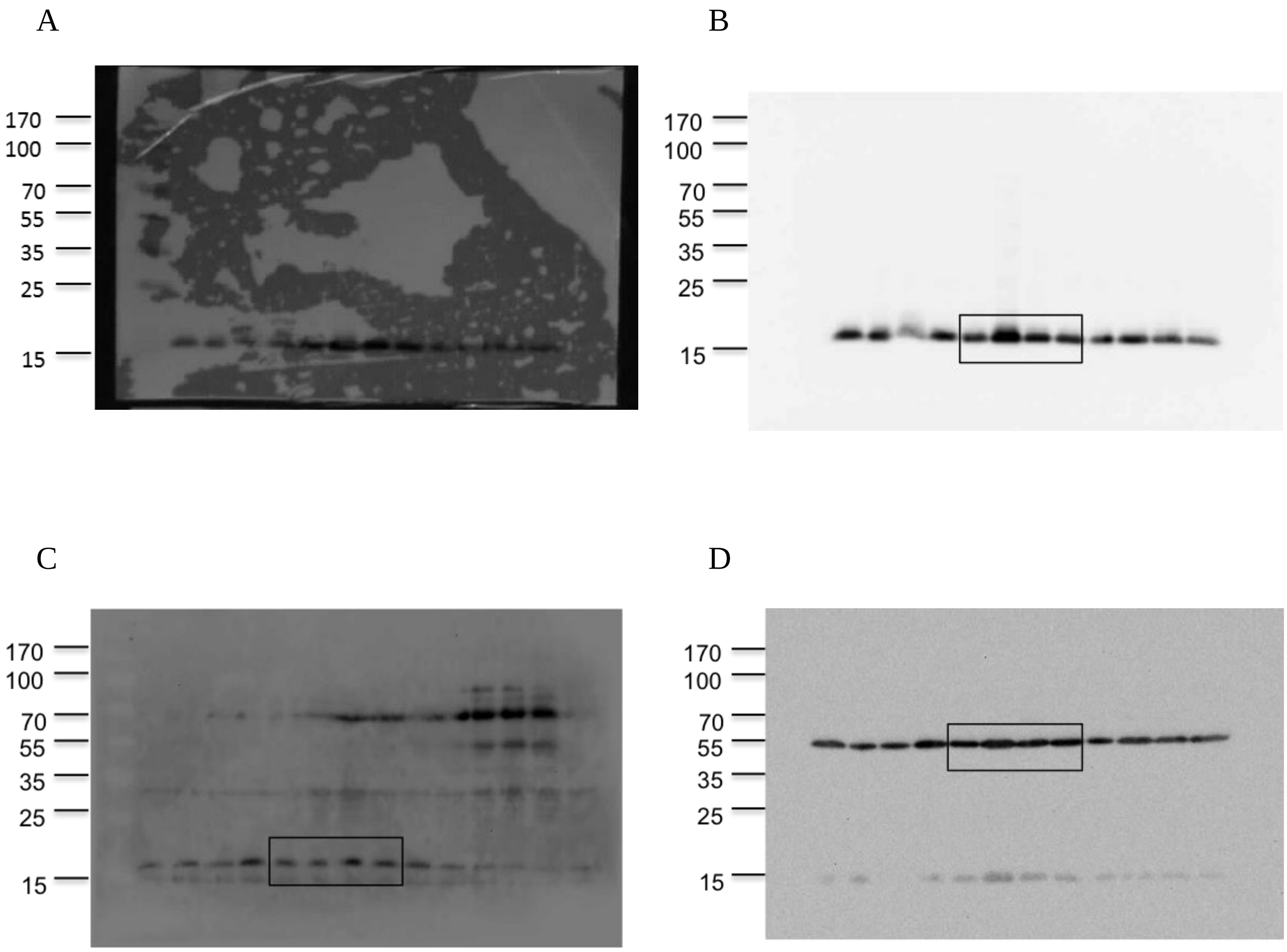

Supplementary Figure 12: Uncropped immunoblots images for Figure 3C displayed in the main figures. Immunoblots are developed using the Syngene gel doc digital dark room system. A digital image of the membrane is acquired, following which, the immunoblot is developed to reveal the probed protein bands (kDa). **A,** A merged image of the captured membrane and developed phosphorylated myosin light chain (MLC) immunoblot. Uncropped immunoblot for **B,** phosphorylated MLC, **C,** total MLC and **D,** β-actin. Boxed areas indicate the cropped regions displayed in Figure 3C.

		Control pre-treated group		HC030031 pre-treated group	
Temperature	Sample Size (n)	Untreated	Temperature-treated	Untreated	Temperature-treated
		Max Change of Blood flow (%)			
4°C	7	-1.5 + 6.0	-32.9 + 5.7 **	-14.6 + 4.4	-45.7 + 3.4 **
10°C	8	-7.4 + 3.4	-34.7 + 3.5 ***	-8.25 + 4.6	-22.0 + 2.9 #
15°C	8	-2.3 + 3.8	-37.4 + 3.9 ***	-8.4 + 5.0	-37.5 + 4.8 ***
18°C	6	3.9 + 3.3	-32.8 + 4.6 ***	-13.4 + 2.7	-39.5 + 2.6 ***
23°C	6	-3.4 + 2.8	-29.6 + 4.0 **	-0.2 + 5.8	-28.3 + 5.7 ***

		TRPA1 WT		TRPA1 KO	
Temperature	Sample Size (n)	Untreated	Cold (10°C)	Untreated	Cold (10°C)
		Max Change of Blood flow (%)			
10°C	4-5	-13.6 + 3.7	-35.9 + 1.9 ***	-1.5 + 9.2	-14.4 + 2.2 #
15°C	5-8	-8.8 + 4.8	-29.5 + 1.2 ***	-1.7 + 4.0	-19.5 + 2.7 *
18°C	6	-2.8 + 2.4	-26.7 + 1.5 ***	-2.3 + 4.2	-23.1 + 1.9 ***
26°C	4	-20.2 + 3.1	-24.7 + 2.6	-8.5 + 8.7	-23.9 + 4.1

Supplementary Table 1: Effects of temperature on vascular blood flow responses. Blood flow was measured in WT mice following local immersion of the ispsilateral hindpaw in water (4, 10, 15, 18, 23 and 26°C) in WT mice pre-treated with the TRPA1 antagonist HC030031 (100mg kg^{-1}, *i.p.*, 30 min) or control (10% DMSO in saline and in TRPA1 WT and KO mice, using Full-Field laser Perfusion Imager (FLPI). Contralateral paw remained untreated. Data is represented % maximum change in hindpaw blood flow from baseline to 0-2 min following local cold treatment (maximum vasoconstriction). All error bars indicate s.e.m *p<0.05, **p<0.01, ***p<0.001 versus respective untreated paw, #p<0.05 versus respective cold-treated hindpaw (ANOVA, Bonferroni *post hoc* test).

Treatment	Sample Size (n)	Blood flow: AUC (10^3 flux units.time)	
		Untreated	Cold (10°C)
Vehicle	5	70.9 ± 9.3	177.1 ± 24.0 ***
SB366791	5	90.8 ± 8.7	185.2 ± 24.2 *
Vehicle	5	71.6 ± 14.8	148.0 ± 20.9 **
AMG9810	5	54.9 ± 11.1	122.9 ± 16.8 *

Transgenic Mice	Sample Size (n)	Blood flow: AUC (10^3 flux units.time)	
		Untreated	Cold (10°C)
TRPV1 WT	7	145.6 ± 12.2	217.8 ± 9.6 ***
TRPV1 KO	8	146.5 ± 11.9	192.3 ± 12.7 *
TRPV4 WT	3	147.3 ± 40.4	258.7 ± 48.5
TRPV4 KO	3	165.7 ± 43.0	272.2 ± 70.6

Supplementary Table 2: Role of TRPV1 and TRPV4 in cold-induced vascular responses. Blood flow response was measured in WT mice following local immersion of the hindpaw in cold water (10°C) in WT mice pre-treated with the TRPV1 antagonist SB366791 (5mg kg^{-1}, *i.p.*, 120 min, n=7) or control (2% DMSO in saline, *i.p.*, 120 min, n=7); TRPV1 antagonist AMG9810 (50mg kg^{-1}, *i.p.*, 30 min, n=5) or control (2% DMSO in saline, *i.p.*, 30 min, n=5); TRPV1 WT (n=7) and KO (n=8) mice and TRPV4 WT and KO mice (n=3) using FLPI. Data represents the total cold-induced restorative response (30 min), as assessed as area under the curve and represented as blood flow (flux units.time). All error bars indicate s.e.m. *p<0.05, **p<0.01, ***p<0.001 versus respective untreated paw (ANOVA, Bonferroni *post hoc* test).

1 Russell, F. A., Fernandes, E. S., Courade, J. P., Keeble, J. E. & Brain, S. D. Tumour necrosis factor alpha mediates transient receptor potential vanilloid 1-dependent bilateral thermal hyperalgesia with distinct peripheral roles of interleukin-1beta, protein kinase C and cyclooxygenase-2 signalling. *Pain* **142**, 264-274, doi:10.1016/j.pain.2009.01.021 (2009).
